# Structural and functional studies of S-adenosyl-L-methionine binding proteins: a ligand-centric approach

**DOI:** 10.1186/1472-6807-13-6

**Published:** 2013-04-25

**Authors:** Rajaram Gana, Shruti Rao, Hongzhan Huang, Cathy Wu, Sona Vasudevan

**Affiliations:** 1 Department of Biochemistry and Molecular Biology, Georgetown University Medical Center, Washington, DC 20007, USA; 2 University of Delaware, 15 Innovation Way, Suite 205, Newark, DE 19711, USA; 3 Department of Biostatistics and Bioinformatics, Georgetown University Medical Center, Washington, DC 20007, USA

## Abstract

**Background:**

The post-genomic era poses several challenges. The biggest is the identification of biochemical function for protein sequences and structures resulting from genomic initiatives. Most sequences lack a characterized function and are annotated as hypothetical or uncharacterized. While homology-based methods are useful, and work well for sequences with sequence identities above 50%, they fail for sequences in the twilight zone (<30%) of sequence identity. For cases where sequence methods fail, structural approaches are often used, based on the premise that structure preserves function for longer evolutionary time-frames than sequence alone. It is now clear that no single method can be used successfully for functional inference. Given the growing need for functional assignments, we describe here a systematic new approach, designated ligand-centric, which is primarily based on analysis of ligand-bound/unbound structures in the PDB. Results of applying our approach to S-adenosyl-L-methionine (SAM) binding proteins are presented.

**Results:**

Our analysis included 1,224 structures that belong to 172 unique families of the Protein Information Resource Superfamily system. Our ligand-centric approach was divided into four levels: residue, protein/domain, ligand, and family levels. The residue level included the identification of conserved binding site residues based on structure-guided sequence alignments of representative members of a family, and the identification of conserved structural motifs. The protein/domain level included structural classification of proteins, Pfam domains, domain architectures, and protein topologies. The ligand level included ligand conformations, ribose sugar puckering, and the identification of conserved ligand-atom interactions. The family level included phylogenetic analysis.

**Conclusion:**

We found that SAM bound to a total of 18 different fold types (I-XVIII). We identified 4 new fold types and 11 additional topological arrangements of strands within the well-studied Rossmann fold Methyltransferases (MTases). This extends the existing structural classification of SAM binding proteins. A striking correlation between fold type and the conformation of the bound SAM (classified as types) was found across the 18 fold types. Several site-specific rules were created for the assignment of functional residues to families and proteins that do not have a bound SAM or a solved structure.

## Background

The post-genomic era is fraught with several challenges, including the identification of the biochemical functions of sequences and structures that have not yet been characterized [1]. These are annotated as hypothetical or uncharacterized in most databases [2,3]. Hence, careful and systematic approaches are needed to make functional inferences and aid in the development of improved prediction algorithms and methodologies. Function can be defined as a hierarchy starting at the level of the protein fold and decreasing down to the level of the functional residues. This hierarchical functional classification becomes essential for annotation of sequence families to a single protein record, which is the mission of the Uniprot Consortium [4]. Understanding protein function at these levels is necessary for translating accurate functional information to these uncharacterized sequences and structures in protein families.

**Figure 1:**
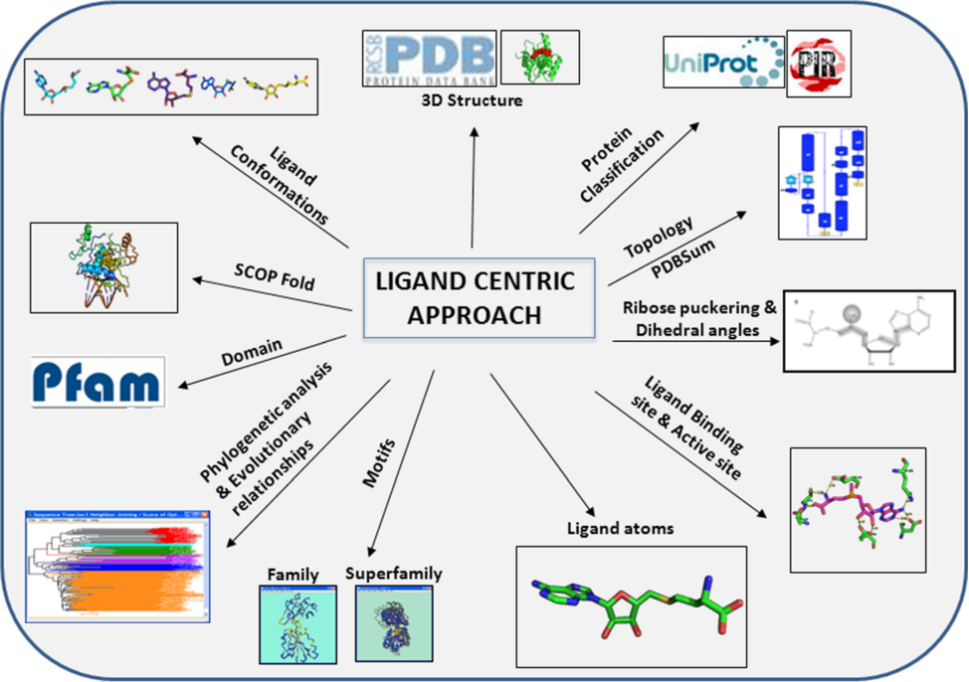
**Ligand-centric approach.** This approach involves a multipronged analysis at various sequence and structural levels. These include analysis at the residue level, analysis at the protein/domain level, and analysis at the family level. At the residue level, this analysis includes identification of conserved binding site residues based on structure-guided sequence alignments of representative members of a family and identification of conserved motifs. At the protein/domain level, analysis includes examination of SCOP folds, pfam domains, and protein topologies. At the ligand level, the analysis includes ligand conformations, ribose sugar puckering (when applicable), and identification of conserved ligand-atom interactions. Finally, at the family level, the approach includes phylogenetic analysis.

Here, we describe a systematic ligand-centric approach to protein annotation that is primarily based on ligand-bound structures from the Protein Data Bank (PDB). Our approach is multi-pronged, and is divided into four levels: residue, protein/domain, ligand, and family levels (Figure 1). Our analysis at the residue level includes the identification of conserved binding site residues based on structure-guided sequence alignments of representative members of a family and the identification of conserved structural motifs. Our protein/domain level analysis includes identification of Structural Classification of Proteins (SCOP) folds, Pfam domains, domain architecture, and protein topologies. Our analysis of the ligand level includes examination of ligand conformations, ribose sugar puckering (when applicable), and the identification of conserved ligand-atom interactions. Finally, our family level analysis includes phylogenetic analysis. Our approach can be used as a platform for function identification, drug design, homology modeling, and other applications. We have applied our method to analyze 1,224 protein structures that are SAM binding proteins. Our results indicate that application of this ligand-centric approach allows making accurate protein function predictions.

SAM, which was discovered in 1952, is a conjugate of methionine and the adenosine moiety of ATP [5]. SAM is involved in a multitude of chemical reactions and is the second most widely used and the most versatile small molecule ligand after ATP [6]. The most well-known biological role of SAM is as a methyl group donor for the covalent modification of a wide variety of substrates, including small molecules, lipids, proteins, DNA, and RNA [7-9]. In addition, SAM is also used as a ligand to transfer other groups that include aminopropyl group transfer in the case of spermidine synthase and tRNA wybutosine-synthesizing protein, ribosyl transfer as in the case of t-RNA-ribosyl transferase isomerase, 5'deoxyadenosyl transfer in 5'fluoro-5'-deoxy-adenosine synthase, and methylene transfer in the case of cyclopropane fatty acid synthase.

Although SAM is widely known to serve as a universal methyl group donor, it is used in the biosynthesis and modification of virtually every class of biomolecule [10]. For example, SAM acts as a precursor in the biosynthesis of nicotinamide phytosiderophores, the polyamines spermine and spermidine, and the plant hormone ethylene. In addition, SAM acts as the source of the 5'-deoxyadenosyl radicals produced as a reaction intermediate by the family of radical SAM enzymes [11,12]. SAM also catalyzes the hydroxylation of the C-10 carbon atom of 15-demethoxy-e-rhodomycin and is involved in the fluorination reactions that take place in some bacteria [13]. Finally, its involvement in binding to RNA riboswitches highlights an interesting connection to the ancient RNA world [14,15].

Because of its important role in many different chemical reactions, SAM has been studied extensively, and its various cellular functions have been described [10,16-18]. Over the past several years, SAM has also become the target of various clinical studies and may have therapeutic value for treating cancer [19,20], Alzheimer’s disease [21], epilepsy [22], depression and dementia [23,24], psychiatric and neurological disorders [25], osteoarthritis [26], and Parkinson’s disease [27]. Thus, computational predictions and methodologies aimed at determining protein function are central to identification of unexplored drug targets, and the results of such methods will most likely aid in the design of drugs to combat these diseases.

## Methods

### Data set

Our analysis included a total of 1,224 structures, of which 666 were ligand-bound. Of these 666, 210 structures had SAM bound, and 456 had S-adenosyl-L-homocysteine (SAH) bound (SAH is the product of the methyl transfer reaction and is structurally equivalent to SAM). The remaining 558 structures were unbound. Data were extracted from the PDB [28], and the PDB-ID codes used are listed in Additional file 1: Tables S1 (column labeled PDB-Ids) for fold type I and Additional file 2: Table S2 for other fold types. The sequence information for the data used in the analysis was extracted from UniprotKB database (http://www.uniprot.org). The 1,224 structures included 16 riboswitches (Additional file 2: Table S2, Sheet labeled riboswitches).

### PIRSF classification

The Protein Information Resource Superfamily (PIRSF) system is built as a hierarchical structure that provides a framework to enable functional annotation at various levels and to cluster full-length proteins into homeomorphic families [29]. Proteins are assigned to the same PIRSF only if they share end-to-end similarity, including similar domain architectures. The 1,224 structures, excluding the 16 riboswitches, were classified into 172 unique families based on clustering analysis (data not shown). One hundred twenty-two of these PIRSFs, as indicated by a unique PIRSF number, have been curated (manually checked and annotated) and are available for download (Additional file 1: Table S1 and Additional file 2: Table S2, column labeled PIRSF). The remaining 50 PIRSFs are in the process of being curated at the Protein Information Resource (PIR) (data not shown).

### Selection of representative structures for analysis

Due to the large number of available structures within the families, one representative SAM/SAH bound structure was chosen from each PIRSF for analysis (Additional file 1: Tables S1 column labeled Representative Protein PDB-ID). The representative structure for each PIRSF was chosen based on three criteria: (a) if multiple SAM-bound structures within a PIRSF existed, the structure with the highest resolution was chosen; (b) if SAM- or SAH-bound structures were available, the SAM-bound structure was chosen; and (c) for PIRSFs that had only unbound structures, the structure with the highest resolution was chosen.

### PIRSF-based site-rules (PIRSR) for fold-type I

The PIRSF classification system provides a platform for the identification of conserved residues in the ligand-binding pocket of a three-dimensional structure. It also allows site-specific features to be assigned to PIRSF members that lack an experimentally determined structure [30]. A SAM/SAH-bound structure, from each of the 111 PIRSFs, belonging to fold type I was chosen as a representative. A structure-guided sequence alignment was constructed using the seed members from each of the PIRSFs using the representative structure as a template. Residues at hydrogen-bonding distance from SAM/SAH were obtained from the PDBsum database [31]. A profile based on the hidden Markov model (HMM) using the HMMER package [32] was created based on the manually edited structure-based alignment. Only residues that were conserved across all members of a given PIRSF were assigned as SAM binding residues and a site-rule was created. This rule was then propagated to other members of the PIRSF that lacked an experimentally determined structure. Structure-guided alignments were created using Cn3d [33] for each of the PIRSF and are available for download upon request.

### Structural fold information

Initial fold information was obtained primarily from SCOP [34]. For structures that did not have any SCOP information, the SUPERFAMILY database that is based on SCOP HMMs [35], was used for structural fold assignment purposes. If no classification existed using either one of the databases, we assigned our own classifications based on manual inspection and other functional attributes (Additional file 1: Table S1, column labeled SCOP fold).

### Topological information

Assignments of the various topological classes were based on the representations from the PDBSum webpage (http://www.ebi.ac.uk/thornton-srv/databases/pdbsum/). The topological class was manually assigned for each of the representative structures. The topology was downloaded and manually labeled (Additional file 1: Table S1, column labeled Topology for fold type I and Additional file 2: Table S2 for other fold types, Additional file 3: Figure S1).

### Sugar puckering

A script was used to generate the various sugar puckering parameters (angle of pseudorotation (P), puckering amplitude V_max,_ out-of-plane pucker and endocyclic torsions ν_0_-ν_4_). In addition to these parameters, the overall conformations of the ligands in terms of their extended or folded nature can be described by the dihedral angles chi and gamma. These definitions follow those of Sun et al. [36]. In addition we define an angle delta. For SAM, Chi is defined as the angle C4-N9-C1’-O4’, gamma is defined as the angle O3’-C4’-C5’-SD, and delta is defined as the angle C4’-C5’-SD-CG. However, the two parameters that adequately describe the sugar pucker are the phase angle of pseudorotation (P) (0º–360º) and the puckering amplitude V_max_ that describes the out-of-plane pucker (Additional file 1: Table S1 and Additional file 2: Table S2, Sheet labeled sugar puckering).

### Ligand superpositions

Different conformations have been observed for the bound ligand within a particular fold type and between different fold types. The liganded structures within each of the classes were superposed using the iTrajComp routine in the Visual Molecular Dynamics (VMD) software package [37]. The ligands were superposed either via their ribose moieties or by using all ligand atoms. For each structure, the resulting r.m.s. deviation was stored as a matrix to be used for further analysis.

### Motifs

Motifs have been previously defined for Rossmann fold MTases**.** These definitions follow Kozbial et al. [16]:

Motif I – The consensus sequence encompassing the N-terminus of the first beta strand and the loop connecting the first beta strand and the adjacent helix.

Motif II – The second beta strand after Motif I.

Motif III – The third beta strand located at the edge of the Rossmann fold.

Motif IV – The fourth beta strand and the flanking loops.

Motif V – The helix following the fourth beta strand.

Motif VI – The motif that corresponds to strand V.

## Results

Here, we have analyzed the 1,224 SAM-binding protein structures currently available in the PDB [28]. Six hundred sixty-six of these structures have SAM/SAH ligands bound to the protein; the remaining are unbound structures. Of the 666 structures, 210 are SAM-bound, and 456 are SAH-bound (SAH is the product of the methyl transfer reaction and is structurally equivalent to SAM). Of the 1,224 structures, 1,208 belonged to 18 different protein folds and the remaining 16 are SAM-dependent riboswitches. Because of the vast amount of data generated upon applying this approach to all 18 fold types, we only discuss the results of fold type I here. The results for the remaining folds are provided additional files. Our approach identified and classified 11 new SAM-binding topologies for the well-studied Rossmann fold MTases. Our approach was also applied to 17 additional SAM binding folds and a striking correlation was observed between fold type and ligand conformations. Finally, our approach resulted in generating functional annotations for 94,640 sequences belonging to 172 SAM-binding families.

**Figure 2:**
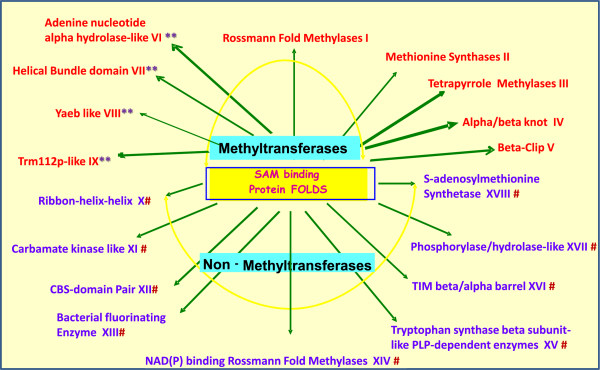
**Fold types of SAM-binding proteins.** The folds follow SCOP classification, except for Helical Bundle, which we have assigned. A total of 18 folds include 9 Mtases and 9 non-MTases indicated by #. Structures belonging to the Rossmann fold methylases have evolved to become MTases and non- MTases and are indicated with a yellow box. SAM-dependent MTases have been previously categorized into five classes by Cheng *et al.*[38]. We have extended this to include a total of nine classes. The four added classes are indicated by **.

The 1,208 structures belonged to 18 different fold types (Figure 2) and 172 homeomorphic families (PIRSFs). These assignments were based on the topological differences that are indicative of the organization of the core strands and helices. Blumenthal et al. [38] defines five classes of SAM-dependent MTases. Based on our four newly identified folds, we extended the Blumenthal et al. classification to include four additional MTase classes. The 18 SAM-bound fold types included 9 MTases and 9 non-MTases. We also defined 14 sub-fold types within fold type I (Table 1).

**Table 1:** Ligfolds and newly classified topological sub-classes

**Topological Arrangement of strands**	**Total number of PDB structures**	**LigFold**	**Topology Subclass**
3214567	351	SAM_DM_Ia	Class Ia
6754123	321	SAM_DM_Ib	Class Ib
32145	2	SAM_DM_Ic	Class Ic
54123	19	SAM_DM_Id	Class Id
564312	29	SAM_DM_Ie	Class Ie
654321	2	SAM_DM_If	Class If
1762354	10	SAM_DM_Ig	Class Ig
7645321	1	SAM_DM_Ih	Class Ih
7654123	12	SAM_DM_Ii	Class Ii
17865234	1	SAM_DM_Ij	Class Ij
5671432	2	SAM_DM_Ik	Class1k
6754123/3214567	1	SAM_DM_Il	ClassIl
3421567	1	SAM_DM_Im	ClassIm
34215687	4	SAM_DM_In	ClassIn

Belong to SCOP fold S-adenosyl-L-methionine dependent methyltransferase (SAM_DM).

### Fold type I and pfam domain distributions (class I): SAM-dependent MTases

Among the available structures, the majority of SAM-binding proteins are MTases that belong to the SAM-dependent MTase fold (also known as the Rossmann fold). This fold type is the best characterized fold type in the MTase superfamily, and is also found in such proteins as spermidine synthases [39], aclacinomycin-10-hydroxylases [40], DNMT2 [41], and a Zn-dependent alcohol dehydrogenase from *Rhodobacter sphaeroides* that bind SAM, but do not possess MTase activity. DNMT2 is recruited for methylation of imprinted genes in germ cells; however, this protein is enzymatically inactive. In addition, non-catalytic Rossmannn fold proteins include mitochondrial transcription factor B (sc-mtTFB) and a t-RNA (1-methyladenosine) MTase from *Saccharomyces cerevisiae*[42,43]. One hundred eleven protein families belong to this fold type (fold type I), and 77 have an assigned PIRSF number; the remaining members are currently being processed (Additional file 1: Table S1 column labeled pfam and PIRSF). These families span a wide variety of proteins whose substrates include small molecules (glycine, histamine, and catechol), RNA (rRNA, tRNA, and mRNA), DNA (adenine, uracil, and cytosine), and proteins (protein-L-isoaspartyl, spermidine synthase, precorrin, and leucine). SAM-binding proteins within fold type I had 75 unique Pfam domain distributions; however three of the families had no domain assignments.

### Topological classes

Most of the fold type I structures are similar and are composed of a basic seven-stranded β-sheet with a central topological switch point and a characteristic reversed β-hairpin at the carboxyl end of the sheet. Our analysis identified several additional topological arrangements. In particular, we observed two major arrangements of the strand topologies within fold type I: those with strand order 3 2 1 4 5 7 6 (commonly reported), and those with strand order 6 7 5 4 1 2 3 (observed in our analysis). Both of these arrangements contain 7 strands that form the core of the β-sheet with the sixth strand running anti-parallel to the other strands. Cyclic permutation of the β-sheets in types Ia and Ib has been reported previously in RNA and DNA MTases, and this alteration is attributed to gene duplication [44].

**Figure 3:**
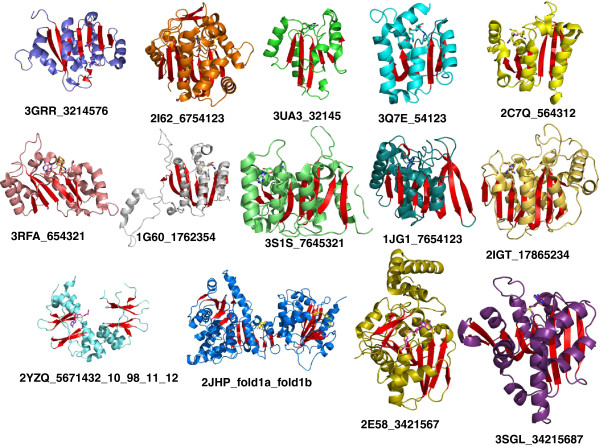
**Topological classes within fold type I.** The classification is based on defining the strands that form the core. The strands are numbered from the N-terminus to the C-terminus and read from left to right. Only the SAM/SAH domain is included. Bound SAM is shown as a ball and sticks, and the structures are represented in cartoon diagrams. The strands that form the core are colored in red. The labels list the PDB-ID followed by the topology. The corresponding two-dimensional topological arrangement is provided in Additional file 3. The figures were generated using PyMOL visualization software (http://www.pymol.org).

To avoid confusion with the existing SCOP folds, we refer to these differing strand order arrangements as sub-types of SAM dependent (SAM_DM) MTase fold and name them as LigFolds SAM_DM_Ia and SAM_DM_Ib, respectively. Of the 1,208 structures, 351 belonged to fold type Ia, and 321 belonged to fold type Ib. In addition, we identified 11 other arrangements of strands with significant deviation from these commonly observed topologies: 5 4 1 2 3 and 3 2 1 4 5 with five strands forming the core; 5 6 4 3 1 2 and 6 5 4 3 2 1 with six strands forming the core; 1 7 6 2 3 5 4, 7 6 4 5 3 2 1, 5 6 7 1 4 3 2, 3 4 2 1 5 6 7 and 7 6 5 4 1 2 3 with seven strands forming the core (but these arrangements deviate from the common Rossmann fold topology); 1 7 8 6 5 2 3 4 and 3 4 2 1 5 6 8 7 with eight strands forming the core. The β-sheet in all of these configurations is flanked by two helices to form a tight αβα sandwich. For clarity, we have defined all of these topologies as sub-types/sub-classes of fold type I (Table 1; Figure 3). The topological classes are provided in Additional file 1: Table S1 (column labeled Topology, Topological Class, Topological sub-class and LigFold).

SCOP classifies all of the above topologies into the SAM-dependent MTase superfamily (Additional file 1: Table S1 column labeled SCOP folds). We suggest classification of the major arrangements into sub-classes, because these different arrangements may have functional consequences. Topological arrangements have previously been shown to be important for identifying the substrate specificities for these enzymes. For example, MTases with small molecules as substrates do not have any C-terminal additions, while MTases with protein substrates contain C-terminal additions [45].

Several structures were not yet classified in SCOP, and in some cases, the SUPERFAMILY database was used, although for several structures, the SUPERFAMILY database yielded only weak hits to unrelated families. In these cases, the structures were manually inspected for classification. For example, the Core Protein VP4 (PDB-ID: 2JHP) had no significant hits at the time of this analysis, but manual inspection revealed that this protein belonged to fold type I and had an interesting topological arrangement comprised of both fold types Ia and Ib (Figure 3). This protein contained two SAM binding sites (one per domain). Topological arrangement 3 2 1 4 5 7 6 (fold type Ia) is inserted between β2 and β3 of the other SAM-binding domain that has the topology 6 7 5 4 1 2 3 (fold Ib). Results of topological analysis for the remainder fold types (II-XVIII) are provided in Additional file 2: Table S2 (column labeled Topology and Topological Class).

### Analysis of ligand temperature factors (B-factors)

**Figure 4:**
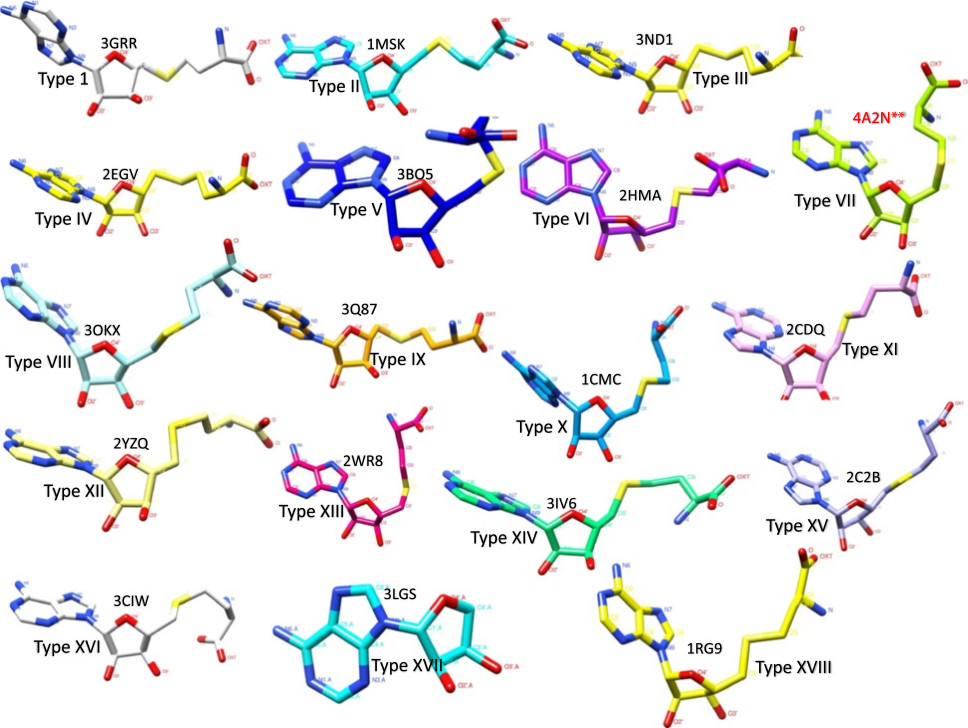
**Ligand conformations across all 18 fold types.** A striking correlation between fold type and ligand conformation was noted. One representative structure was selected from each of the different folds. The structure with the highest resolution was chosen. The ligand SAM/SAH is indicated as a ball and stick. The figure was generated using Chimera visualization software (http://www.cgl.ucsf.edu/chimera/), and atoms are labeled. **Beside Type VII (PDB-ID: 4A2N) indicates an average temperature factor of >80Å^2^ for the ligand and hence may not be reliable. Conformation can be confirmed as more structures become available.

B-factors represent the relative vibrational motion of different parts of a protein structure and its associated ligands. Hence, atoms with low B-factors belong to a well-ordered part of the structure whereas those with high B-factors (> 80 Å^2^) belong to a highly flexible part. To ensure that this flexibility of ligand atoms did not interfere with our ligand conformational and ligand classification analysis, mean temperature factors were calculated for all representative structures. Representative structures with higher temperature factors were flagged and not included in our analysis. Of 666 bound structures, only 23 structures had a mean temperature factor of >80Å^2^. One of the 23 structures that belonged to ligand conformation Type VII (PDB-ID 4A2N) that had a mean temperature factor of >80Å^2^ is included in Figure 4 and is flagged. All structures with average temperature factors higher than 80Å^2^ are also flagged in Additional file 1: Table S1 and Additional file 2: Table S2 (column labeled Temperature Factors).

### Comparisons of ligand conformations across all 18 fold types

**Figure 5:**
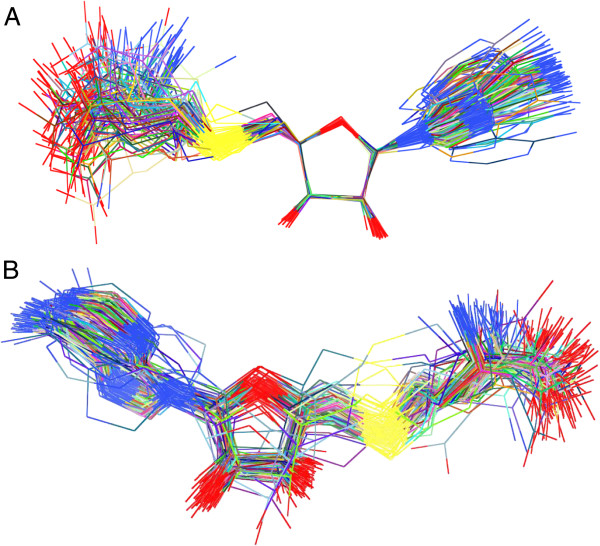
**Superposition of all fold type I SAM/SAH ligands of representative structures from each family that have a mean B-factor of <80Å**^**2**^**. A**. Superposition via the ribose moiety. **B**. Superposition of all SAM atoms. Figure was generated using Chimera Visualization Software (http://www.cgl.ucsf.edu/chimera/).

Ligands from 108 (out of 111) representative structures belonging to the different topological classes within fold type I were compared to a target structure (PDB-ID: 3DLC) via their ribose moieties and by superposition of all ligand atoms (Figure 5A and 5B, respectively). 3DLC was selected as the target because this protein had the highest resolution within fold type I structures. The structures deviated by a mean r.m.s.d. of 1.21 Å when all atoms of the ligands were used for superposition and by 0.067 Å when just the ribose moiety was used for superposition. Three structures were deleted from the analysis as they had a mean temperature factor >80 Å^2^.

An all-against-all comparison of ligand conformations between all fold types (i.e., superposition of all 666 ligand-bound structures that belonged to the 18 different fold types) revealed an interesting and distinctive correlation between fold type and ligand conformation. Because no existing classification of these ligand conformations has been reported, we introduced these different conformations as types (Figure 4).

### Sugar puckering

The existence of the various ligand conformations of SAM and SAH and their correlation with the various fold types emphasize their flexibility. The ligand used in this analysis, SAM, contains adenosine, ribose, and methionine moieties. Ribose is an integral component of many diverse ligands, its pucker and interactions, especially at the O3’ and O2’ positions, are of biological and functional significance [46]. The two parameters that adequately describe the sugar pucker are the phase angle (0º–360º) of pseudorotation (P) and the puckering amplitude (V_max_) that describes the out-of-plane pucker.

The overall conformations of the ligands, in terms of whether they are extended or folded, are dictated by three dihedral angles defined as chi, gamma, and delta as mentioned in the Methods section. For Class I proteins, the majority of the representative structures had a P value between 0º and 180º, although a few exceptions had angles less than 0°. The majority had a distribution of V_max_ in the range 10 to 55. The ribose ring of the ligand predominantly adopted an envelope C1’-exo conformation in 81 cases, a C2’-endo in 10 cases, and an O4’-endo in 10 cases. The C3’-endo and C3’-exo conformations were not commonly observed, except in a few cases. The dihedral angle chi ranged between -140º to +80º, and the gamma and delta angles fell between -180º and +180º. The C3’-endo conformation however were commonly found in fold types II, III, and IV. The results of the analysis for fold type I are provided in Additional file 1: Table S1 (Sheet 2). Results for other fold types are in Additional file 2: Table S2 (Sheet 2). Further analysis is required to establish a relationship between these conformations and substrate specificities.

### Interacting ligand atoms

The goal of this analysis was to identify important interacting SAM atoms with the protein atoms within the context of the various folds. The results of our analysis for representative structures belonging to fold type I are shown in Additional file 1: Table S1 (Sheet 3). The SAM/SAH interactions were predominantly stabilized by H-bonds. The SAM/SAH atoms important for binding were N, N1, and N6 sites of the adenine ring, O2* and O3* sites of the sugar moiety, and the terminal N, O, and OXT atoms. The remaining ligand atoms, N3, N7, N9, SD, and O4*, were rarely found to interact via hydrogen bonds with the protein.

The amino acids often seen interacting at the N-site in all fold type I families were charged residues and small amino acids, that included aspartic acid, glutamic acid, lysine, histidine, tyrosine, and glycine. Hydrophobic residues such as leucine and alanine were occasionally present, but were not commonly found to interact at the N-site. Amino acid residues that interacted at the N1-site included predominantly hydrophobic residues such as leucine, valine, alanine, cysteine, phenylalanine, methionine, and glycine. Amino acid residues that interacted at the N6 site were predominantly charged, with aspartic acid dominating the list of ligand interactions. A few cases, however, interacted with glutamic acid, glutamine, or serine residues. Positions O2* and O3* of the ribose predominantly interacted with charged residues that included aspartic and glutamic acids. O2* and O3* forms the catalytic center of SAM. Not surprisingly, structure-guided alignments of these ligand-interacting residues were conserved in the majority of cases across the PIRSF families, although residues that interacted at positions O and OXT were generally not conserved.

### SAM-binding site

As mentioned earlier, the PIRSF system classifies full-length proteins into homeomorphic families that reflect their evolutionary relationships. Proteins are assigned to the same PIRSF only if they share end-to-end similarity including similar domain architectures (homeomorphic). This system is primarily designed to facilitate the sensible propagation and standardization of protein annotation. Specifically, position-specific rules, or simply site-rules (PIRSR) for annotating functional sites were created manually for all families that have at least one representative ligand-bound structure. Details of the methodology on how rules were created are discussed elsewhere [30]. Briefly, a structure-guided alignment is created for each family, and all of the seed members of a family are aligned to the representative structure of each family. Only residues that were conserved across a family were defined as binding residues, which were then propagated to the rest of the family members that may or may not have a solved structure. Positive matches triggered the appropriate annotation for active site residues, binding site residues, modified residues, or other functionally important amino acids. Additional file 1: Table S1 (column labeled Site rules) lists the residues involved in binding SAM. Only those that were conserved across the family of proteins (based on our structure guided alignments) within a PIRSF for all fold types were included as binding residues. Rules were then created for one representative SAM/SAH-bound structure following the criteria described in the Methods section. One hundred eleven rules were created covering all Class 1 representative structures. Conservative substitutions were observed in many cases. The strict criteria used in this process resulted in high-confidence annotations suitable for incorporation into the Feature Annotations section of UniprotKB.

Although the residues forming the binding pocket were diverse, the shape of the binding pocket itself and the location of the binding pocket were conserved within each fold type irrespective of the different topological classes within fold type I. Based on these rules, functional binding site residues were identified in 94,640 sequences belonging to 122 SAM-binding families (111 belonged to fold type I and 10 to other fold types). Both sequences and structures with and without a ligand were included.

### Structure-guided alignments, CDTree analysis, and motifs

**Figure 6:**
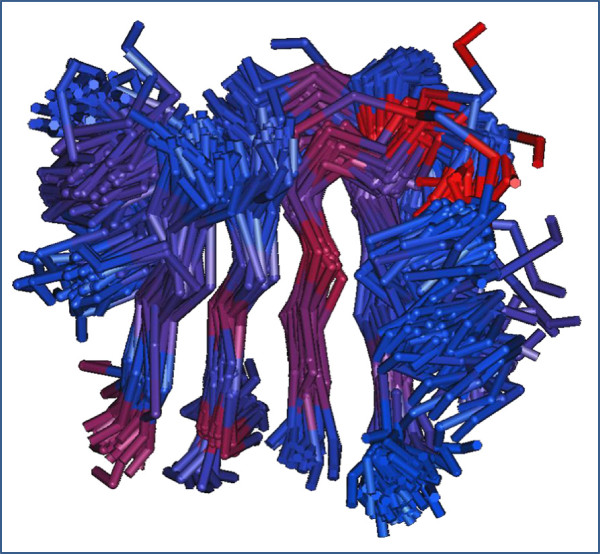
**Structure-guided alignment of representative structures for fold type I.** Only the aligned core is shown. The alignment was completed using the Cn3d tool. The structural representation is shown as tubes.

Structure-guided alignments were carried out with representative members from each of the PIRSFs included in this analysis (Figure 6). Because the sequence identities among the various members are less than <15%, a sequence-based tree will not be meaningful for inferring functional relationships. Hence, a structure-guided alignment of all representative members from the two major topological classes (sub-fold types Ia and Ib) were performed using Cn3d and structural trees were generated using CDTree tool (data not shown). The main goal was to identify sequence and structural motifs.

### Conserved motifs

Several definitions of motifs in MTases have emerged based on the substrates recognized [47-49]. Five regions corresponding to five motifs have been described, and have been shown to occur in the same linear order in the majority of Class 1 MTases. However, for DNA and RNA MTases, a circular permutation occurs after strand 2, and a total of nine motifs have been defined [16,50]. In this paper, we have discussed the five motifs for fold type I (Class I topological classes and sub-classes). The motifs were deduced based on a structure-guided sequence alignment carried out on 111 representative structures from each of the Class I PIRSFs. Two of the motifs (I and II) were conserved in all Class I structures at the superfamily level.

### Motif I (strand I and adjoining loop)

This motif included a consensus GxGxG (G-Glycine) sequence at the N-terminus of the protein, and this sequence was conserved across the entire fold type. The three glycines were conserved in the majority of cases, although a few cases had alanine residues at these positions. This motif was preceded by an invariant acidic residue (Aspartic or Glutamic) at the −2 position from the first glycine and by hydrophobic residues (Leucine, Valine, Isoleucine, Tyrosine, Alanine, or Phenylalanine) at positions −3 and −4 from the first glycine. At least one or two of the three Glycines in the motif interacted with SAM.

### Motif II (strand II and the following helix)

An invariant acidic residue (Aspartic or Glutamic) was present in the middle of strand II and formed a crucial hydrogen bond interaction with the hydroxyls of the ribose moiety of the ligand in majority of the cases. This residue was preceded by hydrophobic residues (Isoleucine, Valine, Phenylalanine, Tryptophan, or Tyrosine) at positions −3 and −4. The helix that followed strand II also contributed to the SAM-binding pocket, especially in fold type Ia with strand arrangement 3 2 1 4 5 7 6. This helix was structurally conserved among all members of this class.

### Motif III (strand III)

A hydrophilic amino acid at the N-terminal end of strand III was present, but was not strictly conserved. This residue was an Aspartic acid in many cases, but other residues such as Serine, Threonine, and Asparagine were sometimes found. In addition, a Glycine was partially conserved at the C-terminal end of this strand. This motif was involved in SAM binding.

### Motif IV (strand IV)

An invariant charged residue, which was usually Aspartic acid, was found closer to the N-terminal end of the strand. This residue was followed by another invariant hydrophobic residue (Valine or Isoleucine) at position +2 from the acidic residue. Also, a second charged residue that is partially conserved was found at the C-terminal end of the strand.

### Motif V (helix following strand IV)

No conserved residues were identified in this motif. In fact, this region is not structurally conserved among the members of this topological class, and this motif was rarely observed to interact with SAM.

### Motif VI (strand V and the preceding loop)

An invariant Glycine residue was found at the beginning of the strand followed by two hydrophobic residues at positions +2 and +3 following the glycine. This motif rarely interacted with SAM.

Although the residues that defined the various motifs themselves were conserved between the two major topological sub-classes, the orientation of the SAM in the binding pocket was different because of the different topological arrangements of the beta strands. In the class with topology 6 7 5 4 1 2 3, motifs I, II, III (in some cases), and IV primarily interacted with SAM. Other motifs only played a minor role in SAM binding. In the sub-class with the 3 1 2 4 5 7 6 topological arrangement, Motifs I (and following helix), II, III, IV, and sometimes V were involved in SAM binding. In neither case was Motif VI involved. In addition to the residues in these motifs, residues in the adjacent loops participate in SAM binding.

### Taxonomic distributions (phyletic patterns) among the various SAM-binding protein families (PIRSFs)

**Figure 7:**
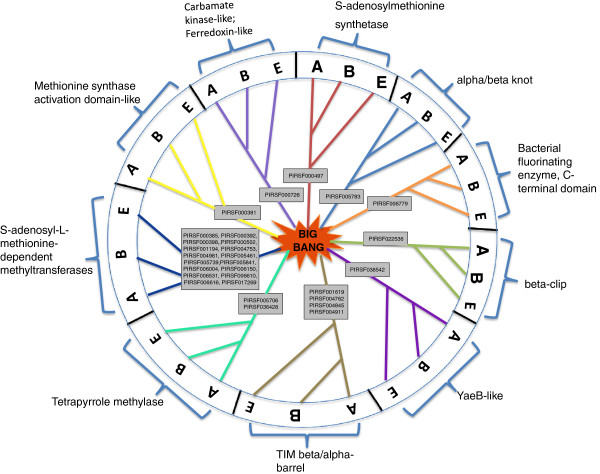
**Taxonomic distributions of SAM-binding proteins.** Families that have representative members from all three branches of life [Archaea (**A**), Bacteria (**B**), and Eukaryotes (**E**)] are indicated within the rectangular boxes. The corresponding fold type is indicated for each of these families along with the circumference of the circle. A total of 29 families that belong to 10 different fold types contain members in all three branches of life. This information may help to identify the last universal common ancestor of SAM-binding proteins.

The analysis presented here is very important for the understanding of the evolution of SAM-binding proteins and for the identification of the Last Universal Common Ancestor (LUCA) of this domain. Although such a discussion is beyond the scope of this manuscript, several review articles that have attempted to trace the evolutionary histories of this domain are available [16,51]. We hope that the data presented in this analysis will aid in further understanding of the evolutionary histories of SAM-binding proteins like which strand arrangement is the most ancient for example. The taxonomic distributions are given in Additional file 1: Table S1 (column labeled Taxonomy). Figure 7 illustrates the divergence of this domain. A total of 29 families that belonged to about 10 different fold types contained representative members from all three branches of life (Archaea, Bacteria and Eukaryotes). One of these likely represents the form of the domain that existed in LUCA.

## Discussion

The goal of our ligand-centric approach is to facilitate discovery of protein function by providing detailed information about ligand binding sites and ligand-specific binding motifs, aiding in structure-based modeling efforts and helping crystallographers identify unexpected molecular commonalities and similarities with other protein-ligand systems.

Carrying out comparative analysis on binding sites of similar ligands yields valuable information about conserved and non-conserved interactions. While the conserved interactions are determinants of ligand affinity, the non-conserved interactions govern the specificity. For example, similarities between the ligand binding sites of an odorant receptor and metabotropic glutamate receptors defined the motif for ligand recognition in the G-protein coupled receptor superfamily [52]. Our ligand conformational and classification analysis will aid in choosing the right conformation of the ligand for docking studies. For example, if only an unbound structure exists, one can presumably pick the correct conformation based on its fold and ligand type to dock the appropriate conformer into the binding pocket. This information can play an important role in future drug design.

Our in-depth analysis of the fold types revealed some unexpected findings and several new classes within fold type I. It also allowed us to identify other new SAM-binding folds (Figure 2). We found a unique case of a histone-lysine N-MTase within the Rossmann fold family that specifically methylates histone H3 to form H3K79me (DOT1). This is surprising because the majority of the histone methylases belonged to the beta-clip fold (Class V, fold type V). However, this family of MTases lacks the traditional SET domain that is found in the majority of the histone MTases [53,54]. This suggests that this family of proteins have evolved an alternative mechanism for histone methylation that is specific to fungi and is involved in telomere silencing [55]. Histone MTases and demethylases have rapidly emerged as epigenetic modifiers that offer new and promising classes of therapeutic targets [19,20]. Other fold types in our analysis do not exhibit as much diversity in substrates as fold type I. For example, fold type II predominantly included protein MTases, fold type III included tetrapyrrole methylases, fold type IV included RNA methylases, and fold type V included the SET domain-containing histone methylases.

Our methodology was recently used for SAM-binding site prediction in Tyw2, an enzyme in the human wybutosine pathway. The binding site residues were predicted based on the created rules and these were experimentally verified [56]. Our study identified important ligand atoms that differentiate methyl transfer and aminopropyl transfer. The rigor in our methodology renders high-confidence annotations. For example, Table 2 provides examples of unbound SAM dependent structures. These structures are all annotated as structures of unknown function. While simple homology-based methods might reveal that these are MTases, our approach can with high confidence predict the binding site (based on family structure guided alignments), type of ligand-conformation, topological class, taxonomic distributions, and a better protein name that reflects its function. Our analysis will also enable prediction of substrate specificities based on the topological arrangements of the strands and sugar pucker as described earlier.

**Table 2:** Annotation of uncharacterized proteins based on our ligand-centric approach

**PDB ID**	**CURRENT ANNOTATION IN PDB**	**PIRSF ID**	**TAXONOMY**	**PREDICTED LIGAND CONFORMATION & CLASS**	**SUGGESTED NAME**
2PGX	Crystal structure of UPF0341 protein yhiQ from E. coli	SF016106	E=0, B=156, A=0, V=0, O=4	Type 1	Putative SAM dependent r-RNA methyltransferase
				Class 1a	
2O3A	Crystal structure of a protein AF_0751 from Archaeoglobus fulgidus	SF016123	E=0, B=0, A=134, V=0, O=0	Type IV	Putative SAM dependent t-RNA archaeal methyltransferase
				Class IV	
2B78	A putative sam-dependent methyltransferase from Streptococcus mutans	SF004981	E=2, B=403, A=16, V=0, O=5	Type 1	Putative SAM dependent RNA methyltransferase
				Class 1a	
3DR5	Crystal structure of the Q8NRD3_CORGL protein from Corynebacterium glutamicum	SF005841	E=122, B=346, A=6, V=0, O=3	Type 1	Putative SAM dependent COMT type methyltransferase
				Class 1b	
1XXL	The crystal structure of YcgJ protein from Bacillus subitilis at 2.1 A resolution	SF006616	E=6, B=130, A=2, V=0, O=4	Type 1	Putative SAM dependent Class Ib methyltransferase
				Class 1b	
1YB2	Structure of a putative methyltransferase from Thermoplasma acidophilum	SF017269	E=227, B=418, A=110, V=0, O=2	Type 1	Putative SAM dependent t-RNA methyltransferase
				Class 1a	
1JSX	Crystal Structure of the Escherichia coli Glucose-Inhibited Division Protein B (GidB)	SF003078	E=19, B=4040, A=0, V=0, O=13	Type 1	Putative SAM dependent r-RNA methyltransferase
				Class 1b	

Functions are assigned based on the results of the analysis presented in this manuscript. Majority of the structures are from Structural Genomics Initiatives with unassigned functions.

Systematic examination of proteins using this approach will unravel structural determinants of enzyme catalysis and facilitate the definition of a toolkit that is specific for these families of proteins. The data presented in this manuscript will be made available *via* the LigFam database. The LigFam database itself will be discussed in a future manuscript. LigFam has powerful search engines to retrieve any information on SAM that has been described here. In addition, we have applied our ligand-centric approach to other ligands that include Nicotinamide-adenine-dinucleotide (NAD), Adenosine-5'-triphosphate (ATP), Guanosine-5'-triphosphate (GTP), Guanosine-5'-diphosphate (GDP) and pyridoxal-L-phosphate (PLP) which will be discussed elsewhere.

## Conclusion

Our ligand-centric analysis has enabled identification of new SAM-binding topologies for the most well studied Rossmann fold MTases and many topological classes. A striking correlation between fold type and the conformation of the bound SAM was noted (classified as types), and several rules were created for the assignment of functional residues to families and proteins that do not have a bound SAM or a solved structure (which we designate site rules). These rules and results of the ligand-centric analysis will enable propagation of annotation to about 100,000 protein sequences that do not have an available structure.

Our method is limited by the availability of structures with bound ligands. In particular, we may be missing some important functional relationships that may be evident in unbound structures.

## Authors’ contributions

Conceived the idea and designed the analyses: SV. Performed the analyses and collected data: SV, RG, SR. Data analyses: RG, SV. Contributed analyses tools: HH, WUC. Wrote the manuscript: SV, RG. All authors read and approved the final manuscript.

## Financial competing interests

In the past five years have you received reimbursements, fees, funding, or salary from an organization that may in any way gain or lose financially from the publication of this manuscript, either now or in the future? Is such an organization financing this manuscript (including the article-processing charge)? If so, please specify. **No.** Do you hold any stocks or shares in an organization that may in any way gain or lose financially from the publication of this manuscript, either now or in the future? If so, please specify. **No**. Do you hold or are you currently applying for any patents relating to the content of the manuscript? Have you received reimbursements, fees, funding, or salary from an organization that holds or has applied for patents relating to the content of the manuscript? If so, please specify. **No.** Do you have any other financial competing interests? If so, please specify. **No.**

## Non-financial competing interests

Are there any non-financial competing interests (political, personal, religious, ideological, academic, intellectual, commercial or any other) to declare in relation to this manuscript? If so, please specify. **No.**

## Supplementary Material

Additional file 1: Table S1 Results of ligand-centric analysis for fold type I. Click here for file

Additional file 2: Table S2 Results of ligand-centric analysis for other fold types (II-XVIII). Click here for file

Additional file 3: Figure S1 Topological diagrams for the various subclasses identified for fold type I. Click here for file
