# Supplementary figures and images for: Structural and functional studies of S-adenosyl-L-methionine binding proteins: a ligand-centric approach
Source: BMC Struct Biol. 2013 Apr 25;13:6. doi: 10.1186/1472-6807-13-6 (PMC3662625; doi:10.1186/1472-6807-13-6)

3UA3

32145

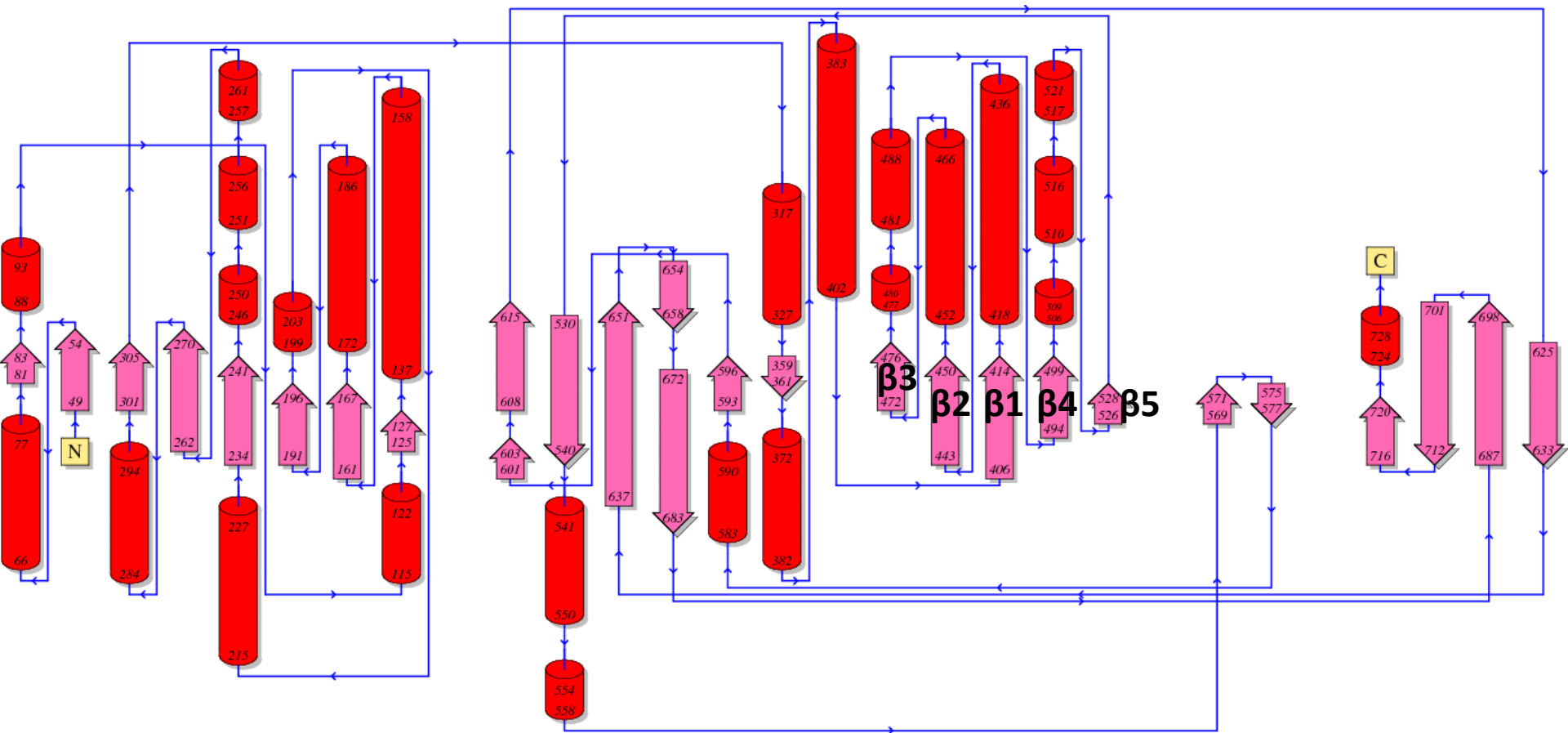

3q7e

54123

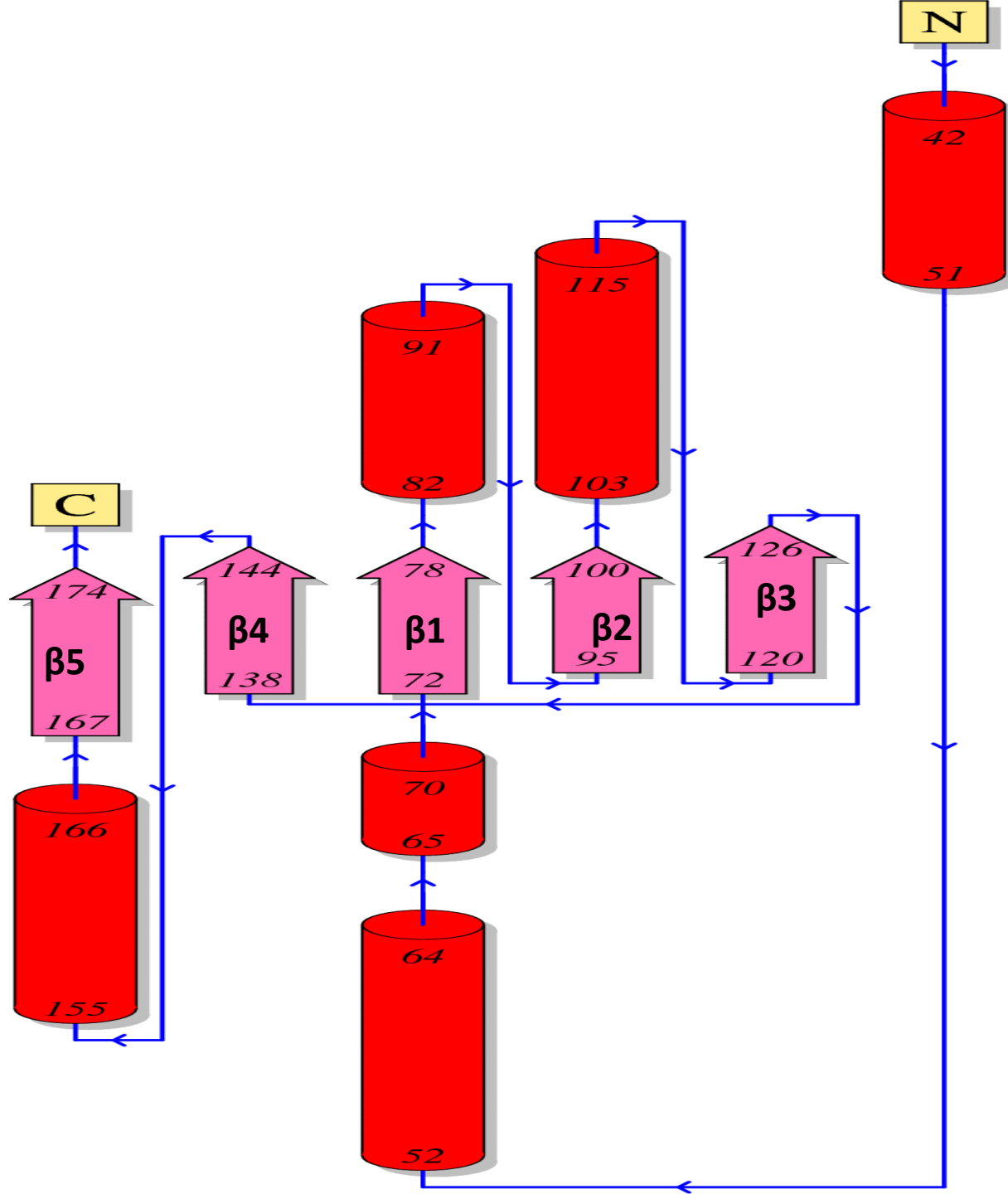

1G55

3214576

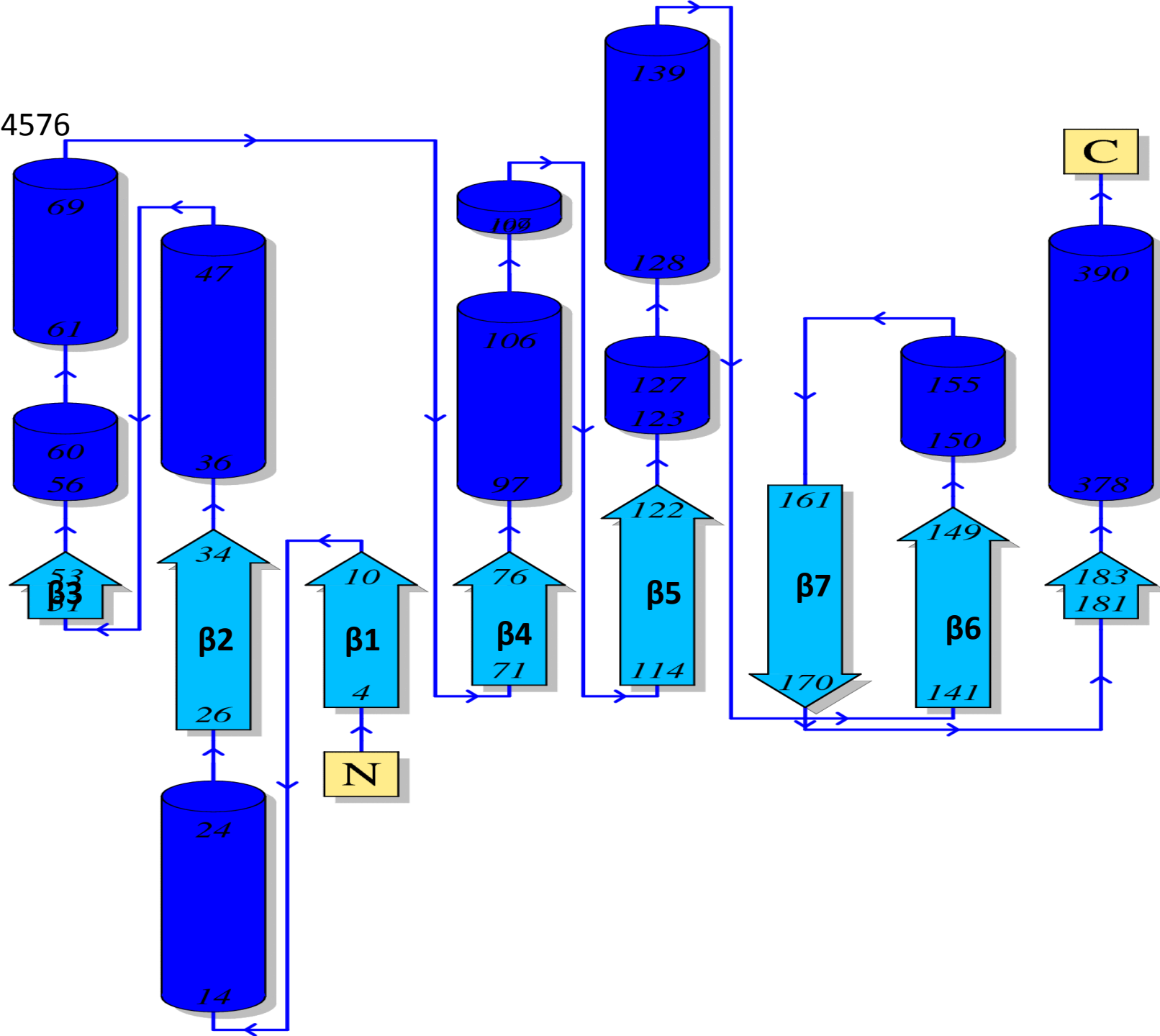

2C7Q  
564312

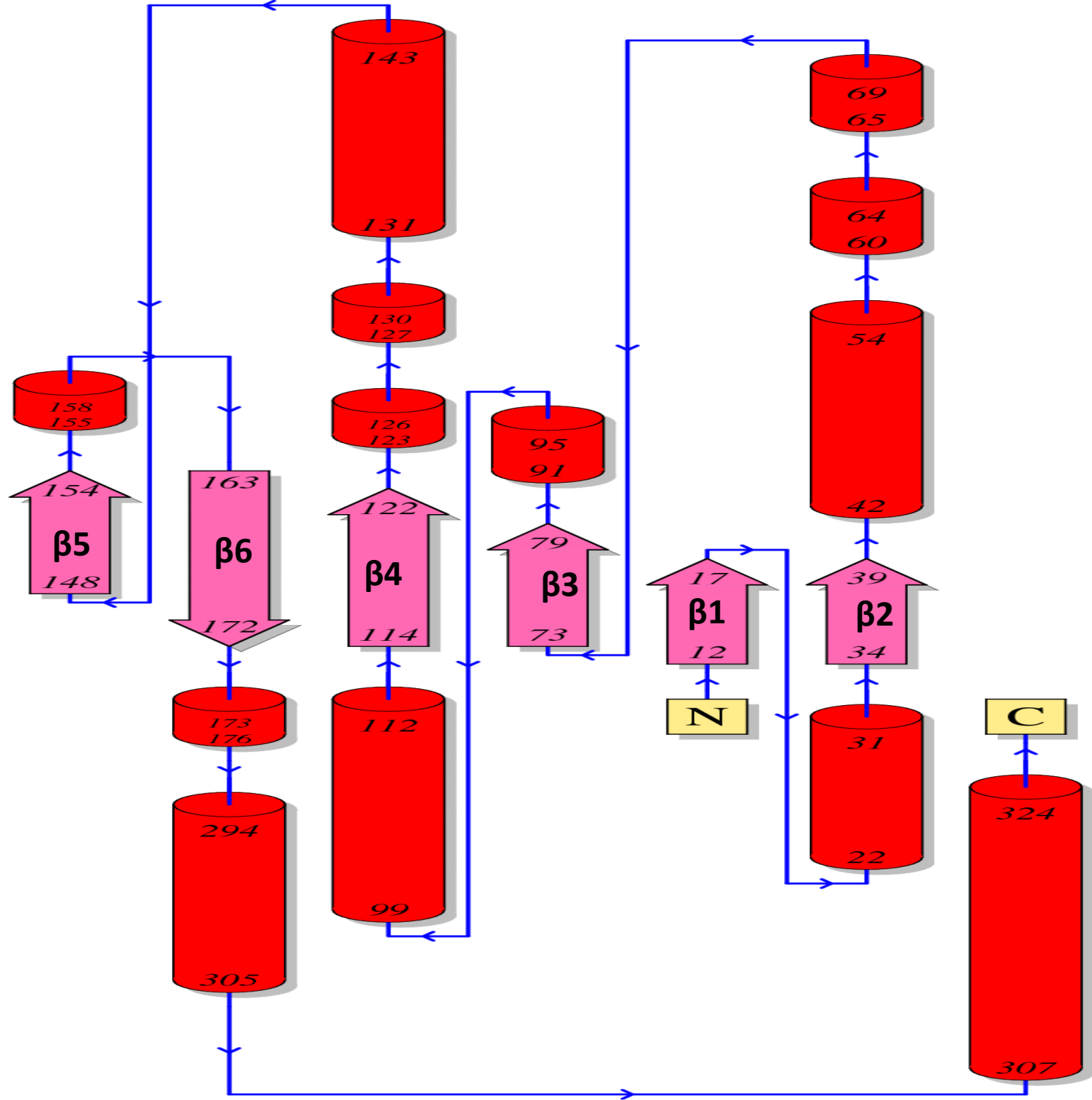

3RFA 654321

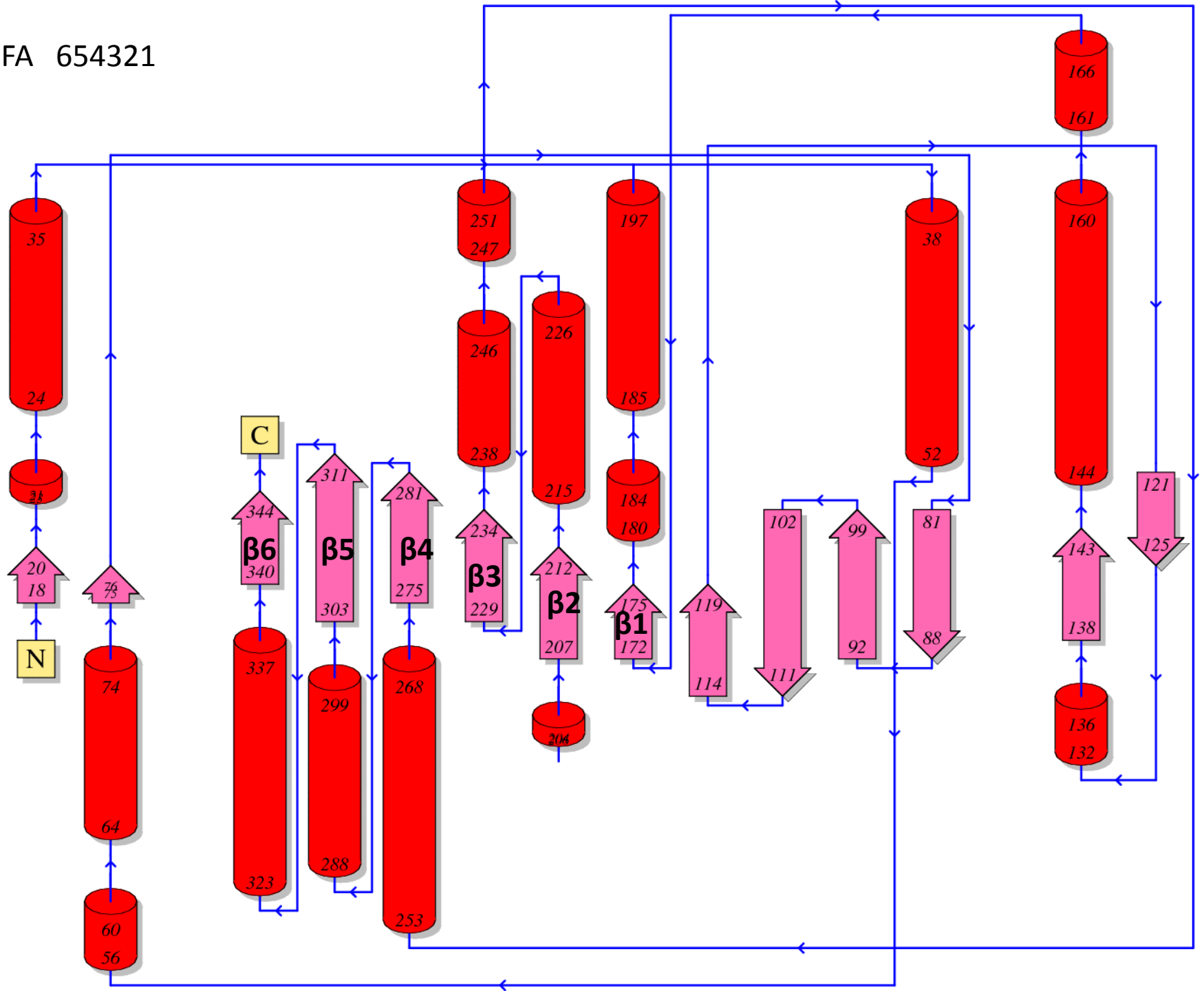

1G60

1762354

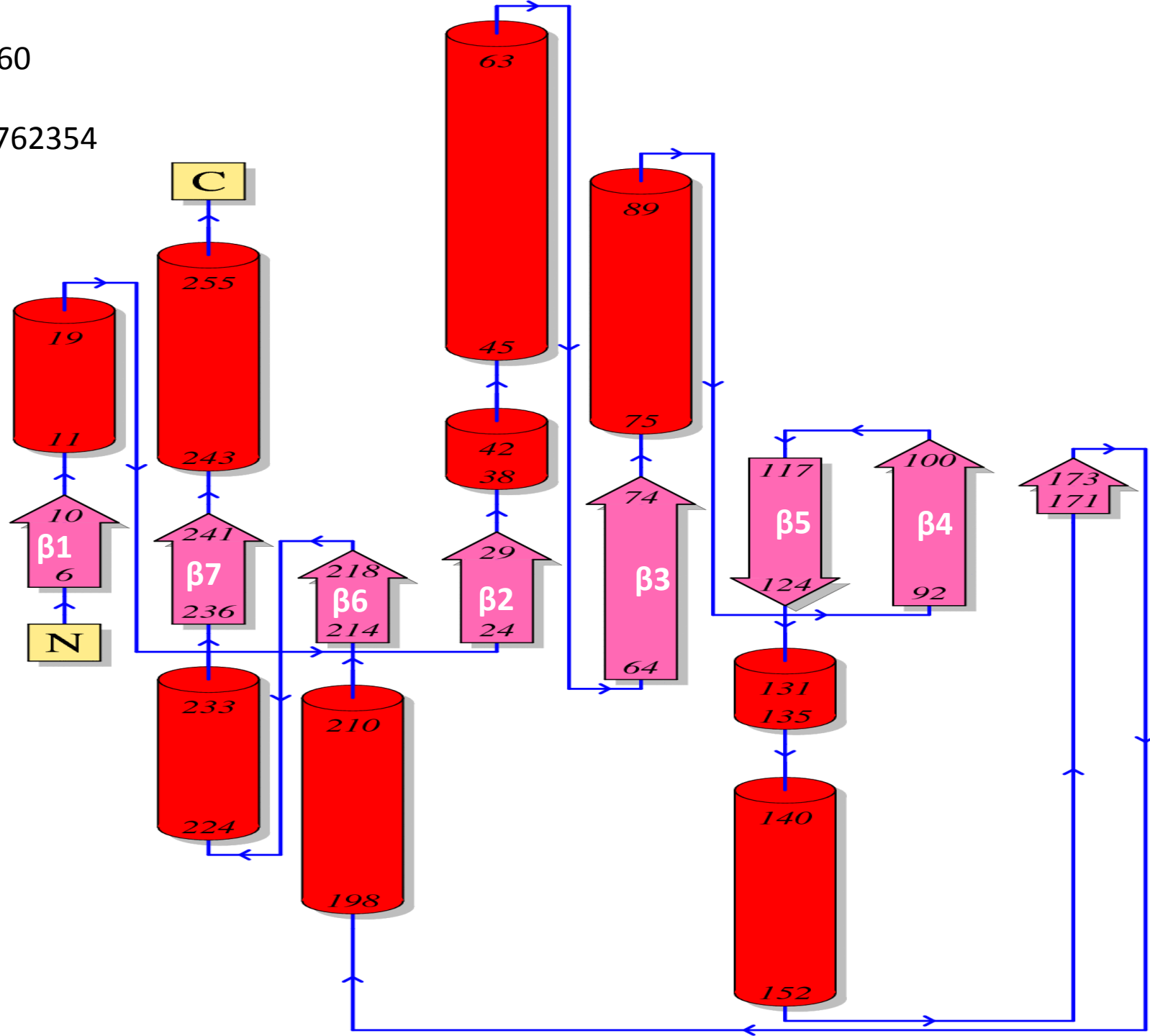

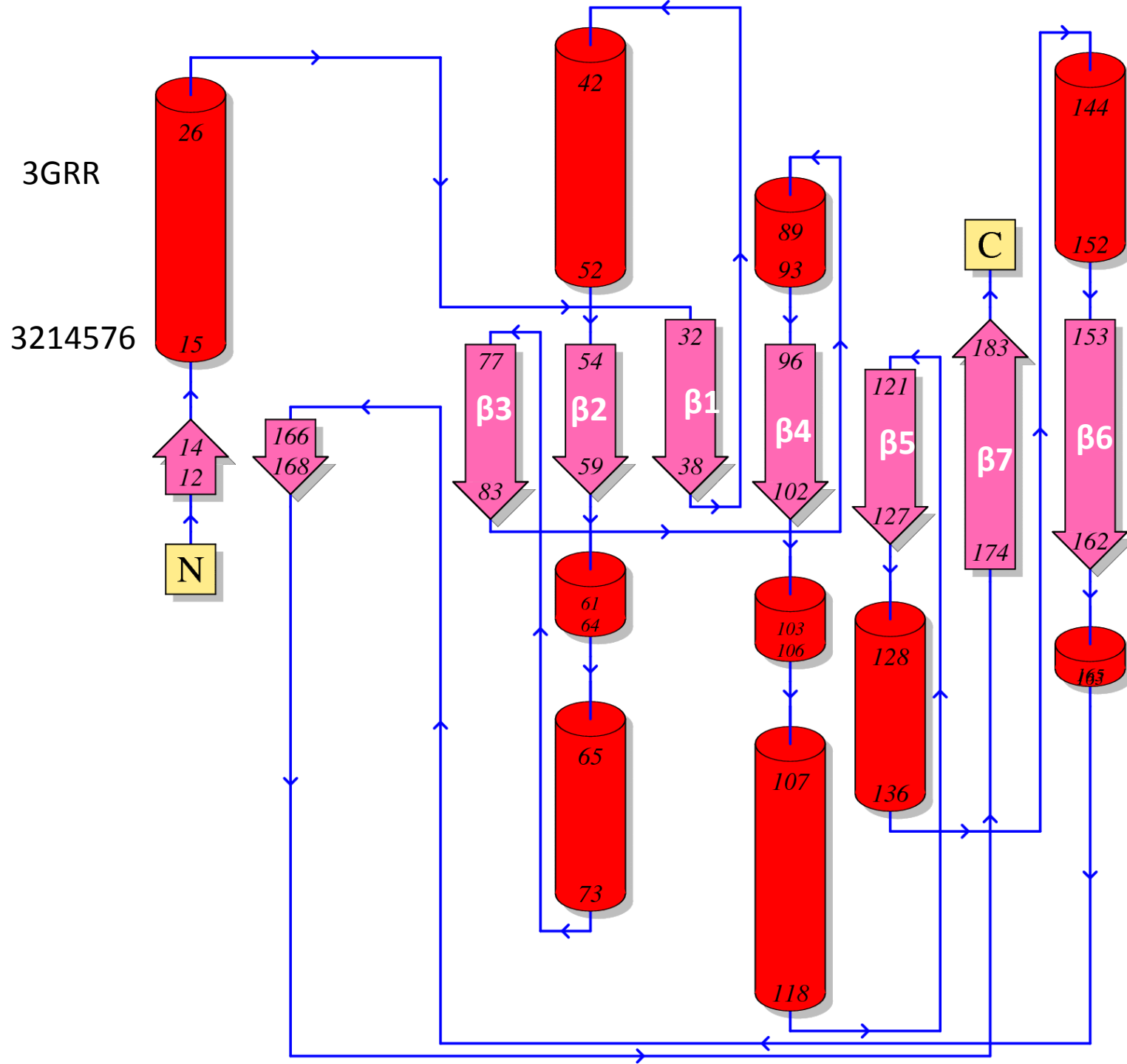

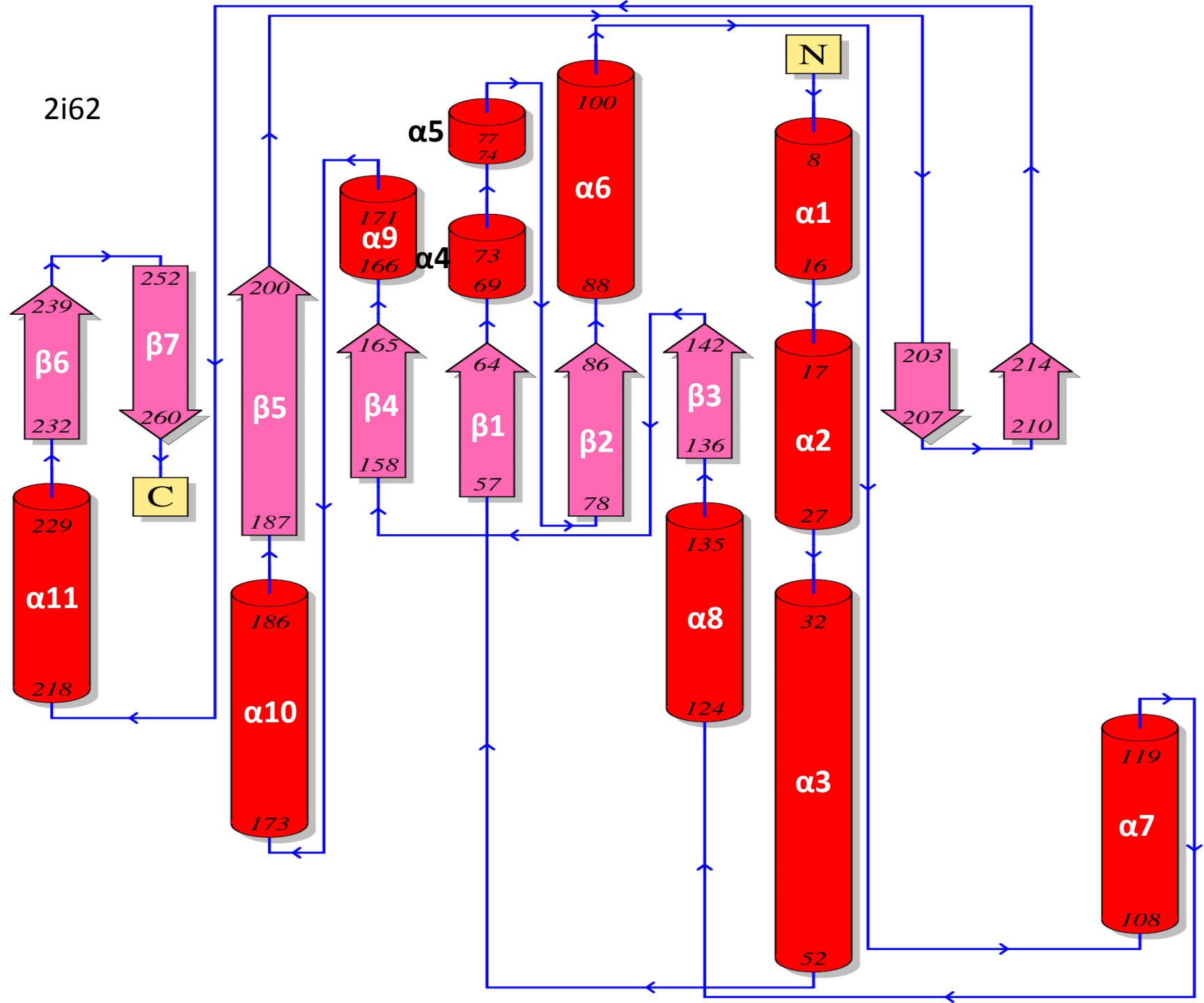

3S1S

7645321

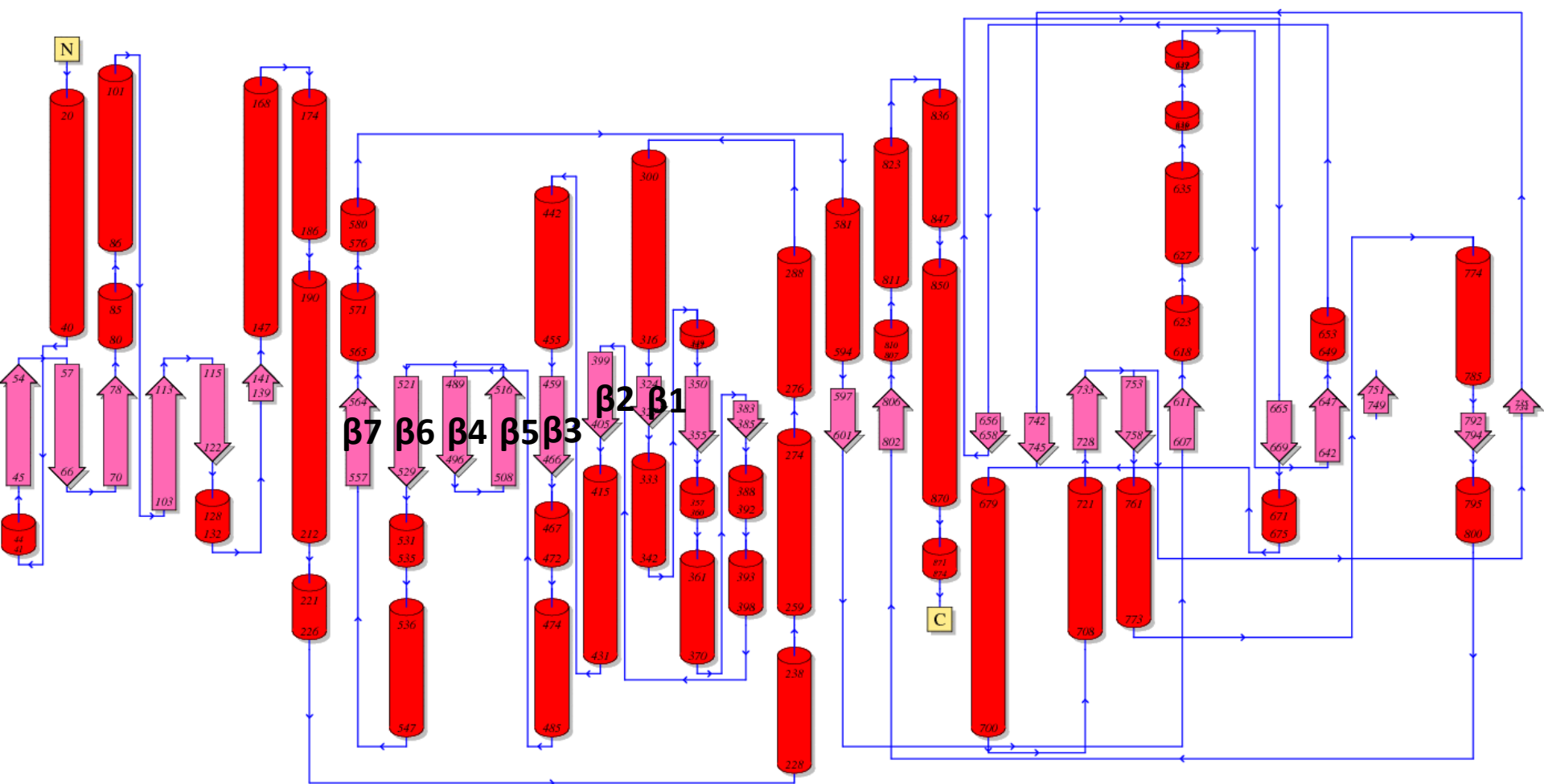

1JG1  
7654123

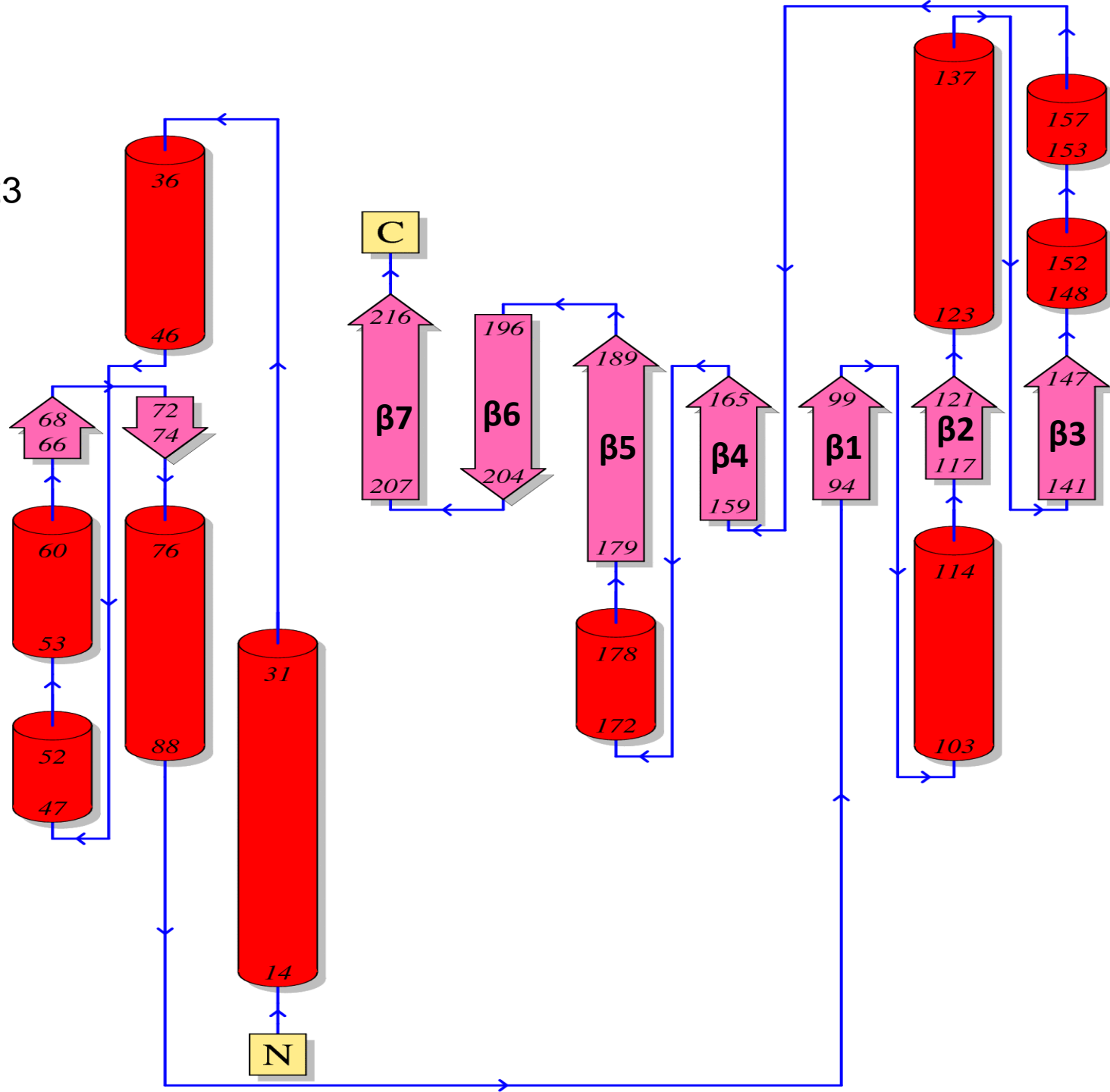

2IGT  
17865234

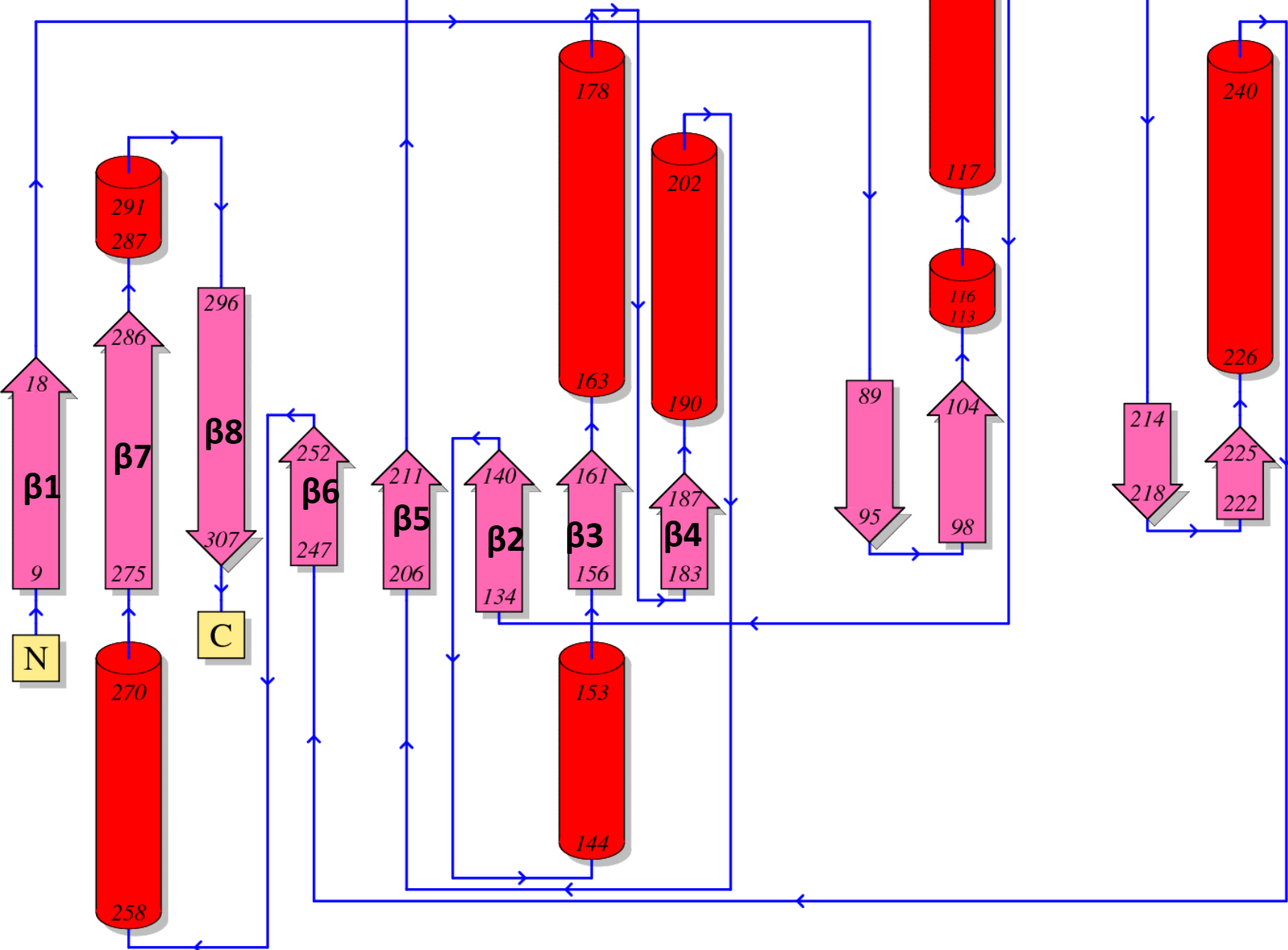

17865234

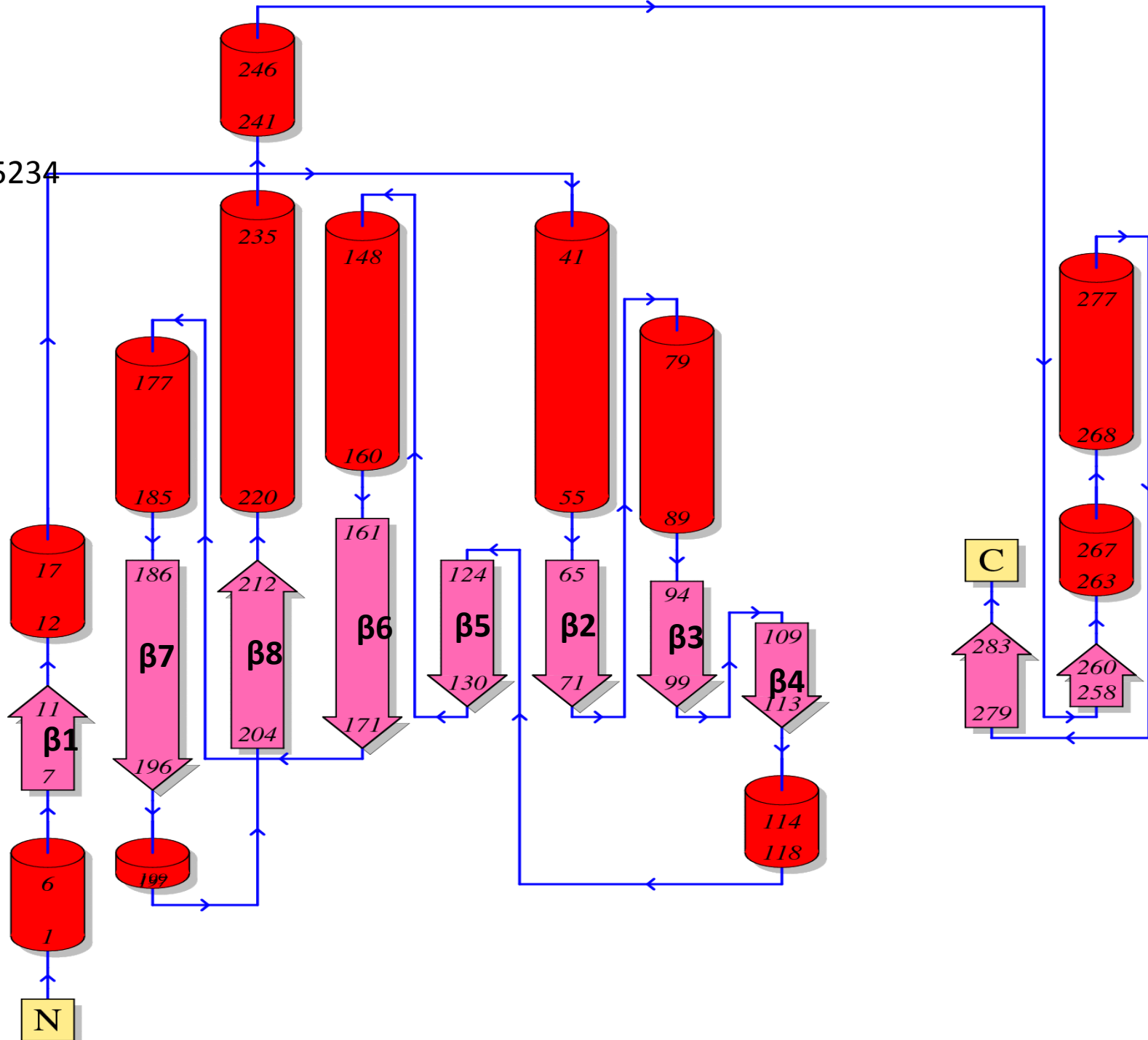

3SGL  
34215687

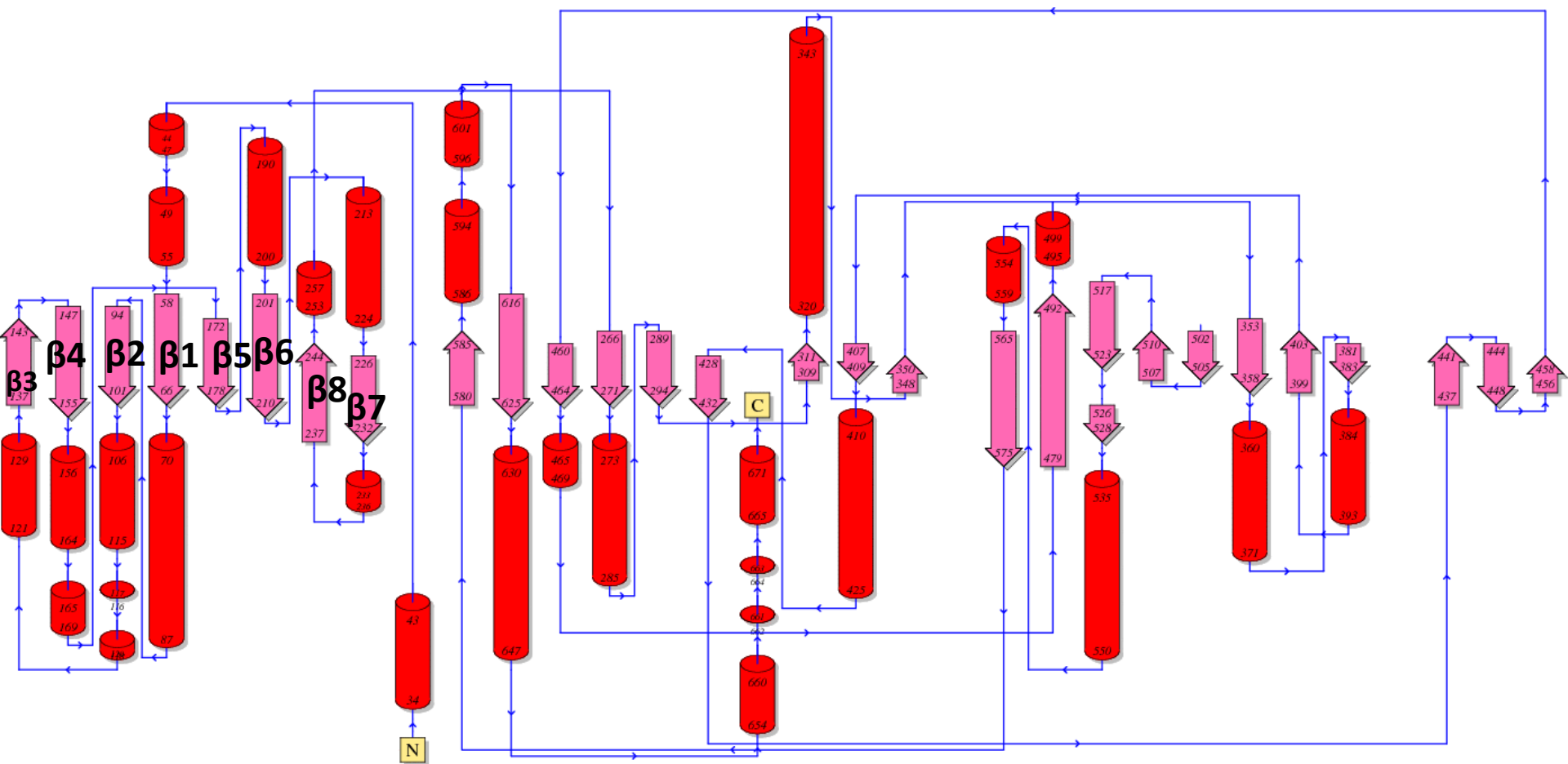

2yzq  
5671432 10 98 11 12

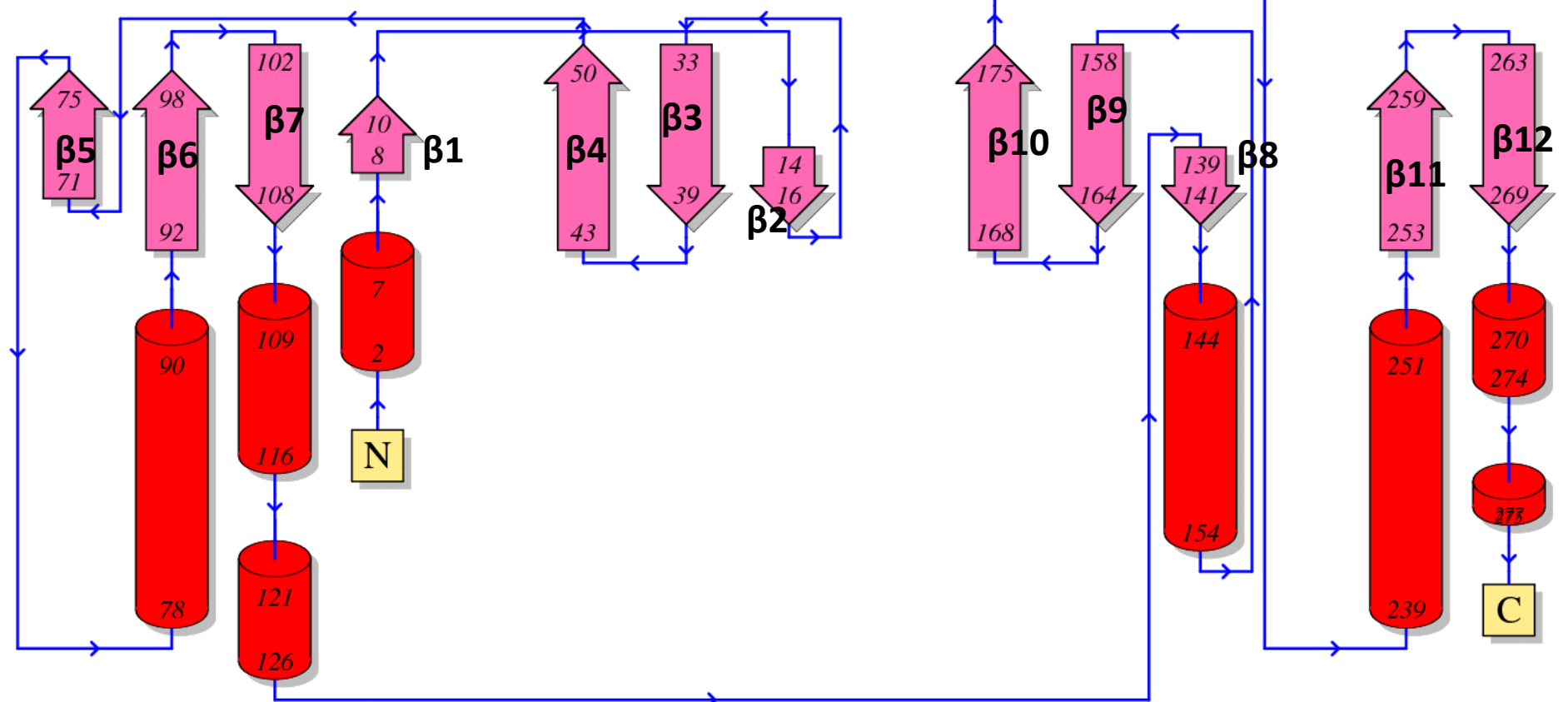

6754123

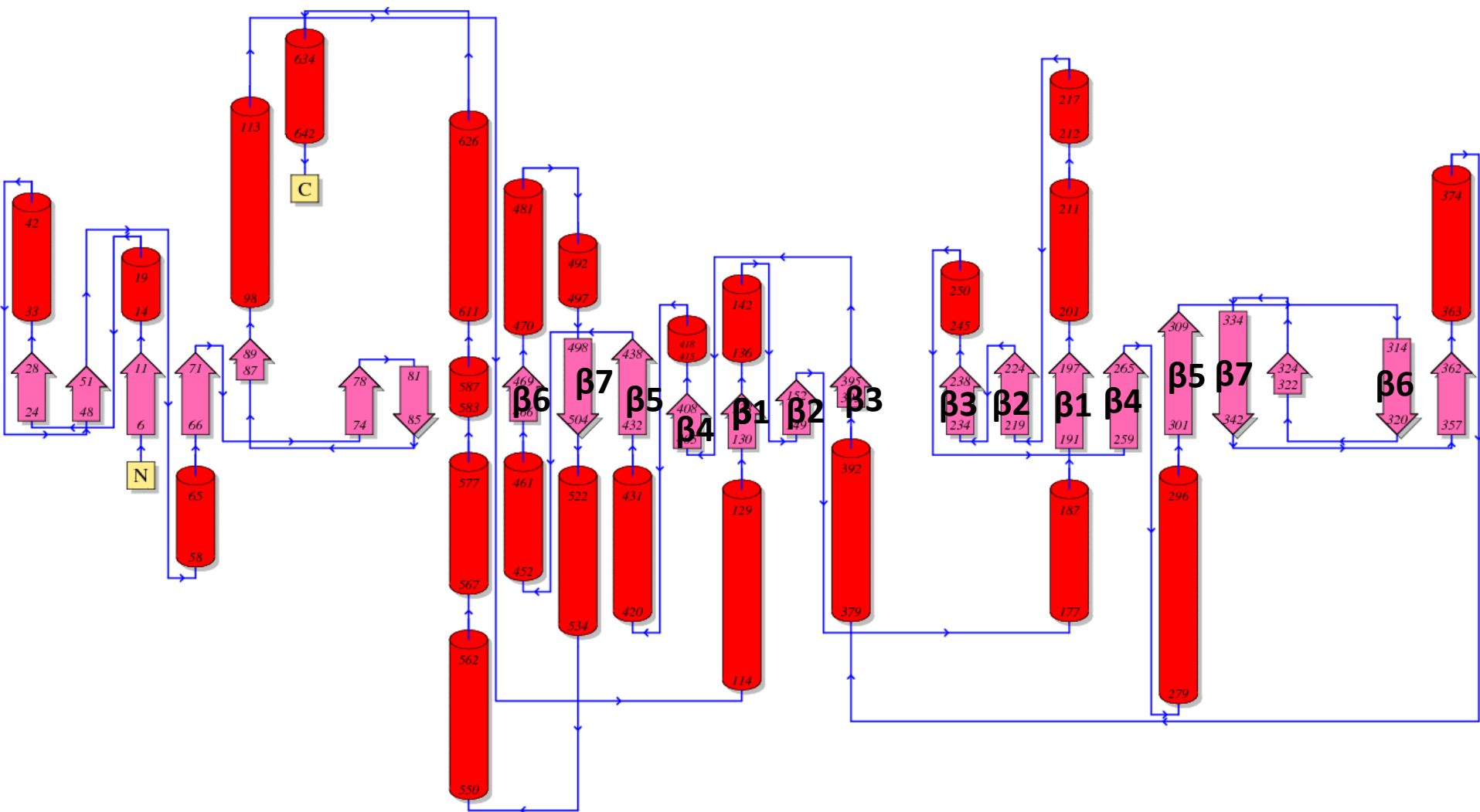

2E58

3421567

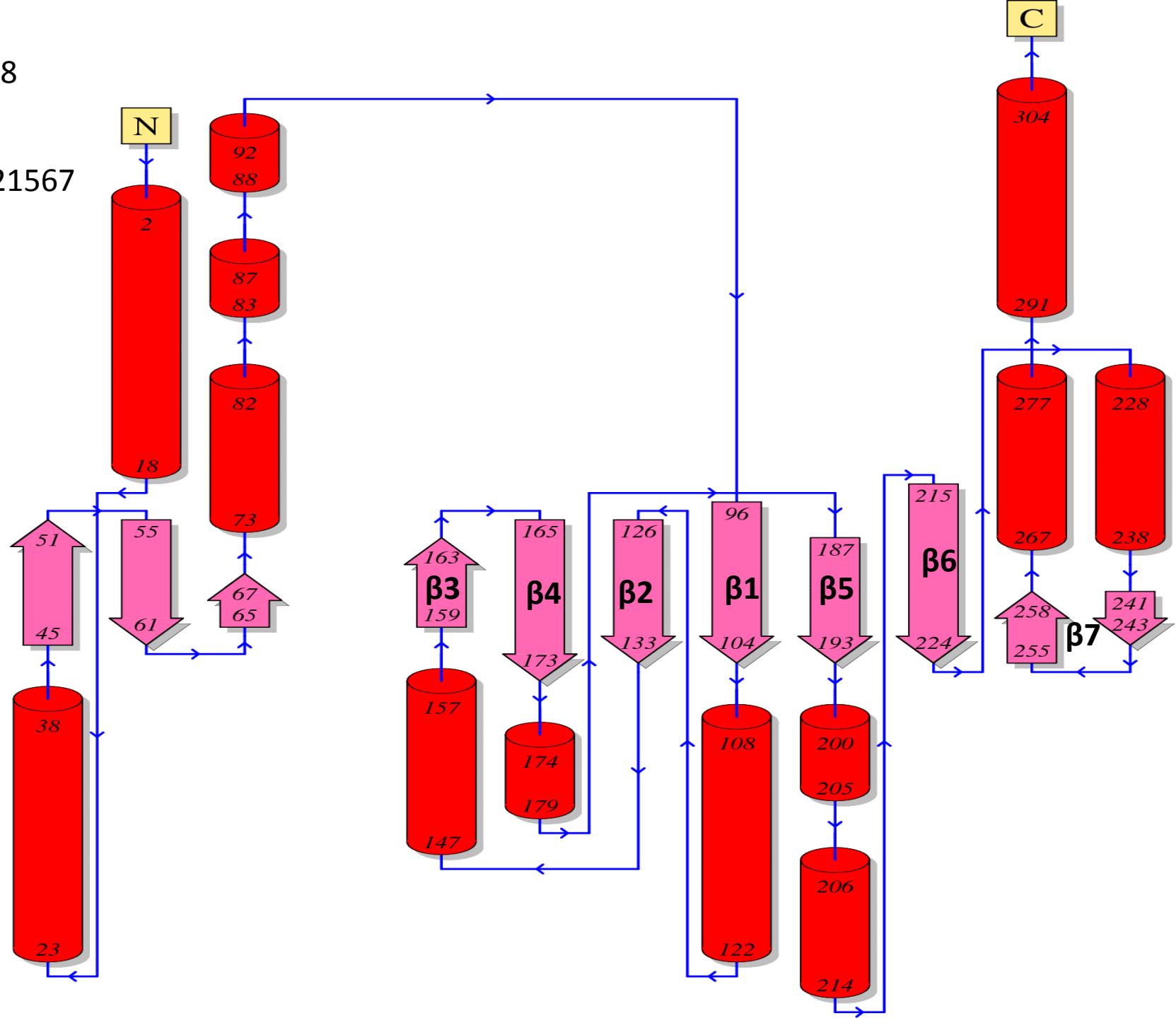

Supplement: Additional file 3: Figure S1 — Topological diagrams for the various subclasses identified for fold type I. [file 1472-6807-13-6-S3.pdf]
